# Barriers and facilitators towards implementing the Sepsis Six care bundle (BLISS-1): a mixed methods investigation using the theoretical domains framework

**DOI:** 10.1186/s13049-017-0437-2

**Published:** 2017-09-19

**Authors:** Neil Roberts, Guy Hooper, Fabiana Lorencatto, Wendell Storr, Michael Spivey

**Affiliations:** 1 Departments of Critical Care and Anaesthesia, Royal Cornwall Hospital, Truro, Cornwall, TR1 3LJ UK; 2 Centre for Health Services Research and Management, School of Health Sciences, City University of London, London, UK

**Keywords:** Sepsis, Theoretical domains framework, Barriers, Facilitators, Quality improvement, Implementation

## Abstract

**Background:**

The ‘Sepsis 6’, a care bundle of basic, but vital, measures (e.g. intravenous fluid, antibiotics) has been implemented to improve sepsis treatment. However, uptake has been variable. Tools from behavioral sciences, such as the Theoretical Domains Framework (TDF) may be used to understand and address such implementation issues. This study used a behavioral science approach to identify barriers and facilitators towards Sepsis Six implementation at a case study hospital.

**Methods:**

Semi-structured interviews based on the TDF were conducted with a sample group of consultants, junior doctors and nurses from Emergency Department, Medical and Surgical Admissions, to explore barriers/facilitators to Sepsis Six performance. Transcripts were analyzed following the combined principles of content and framework analysis. Emerging themes informed a questionnaire to explore generalizability and importance across a sample of 261 stakeholders. Median importance and agreement ratings for each theme were calculated overall and for each role and clinical area. These were used to identify important barriers and important facilitators as targets for performance improvement.

**Results:**

No new belief statements were discovered and data saturation was deemed achieved after 10 interviews. 1699 utterances were coded into 64 belief statements, then collated into a 51-item questionnaire. 113 questionnaire responses were obtained (44.3% response rate). Important barriers included insufficient audit and feedback, poor teamwork and communication, concerns about using the Sepsis Six in certain patients, insufficient training, and resource concerns. Facilitators included confidence in knowledge and skills, beliefs in overall benefits of the bundle, beliefs that identification and management of septic patients fell within everyone’s role, and that regular use of the bundle made it easier to remember. Some beliefs were applicable for the entire group, others were specific to particular staff groups.

**Discussion and Conclusions:**

A range of barriers and facilitators towards Sepsis Six performance across different staff groups were systematically identified using a theoretically-informed approach. This can inform development of targeted performance improvement interventions.

**Electronic supplementary material:**

The online version of this article (10.1186/s13049-017-0437-2) contains supplementary material, which is available to authorized users.

## Background

Sepsis remains a global issue, with timely recognition and treatment crucial to outcome. Recent international consensus emphasizes importance of early identification of organ dysfunction in patients with infection [1]. Timely antibiotic administration in septic patients has been adopted as a national standard against which UK hospitals are measured, with performance linked to financial reward or punishment [2]. Indeed, modern care standards, with early antibiotics and fluid resuscitation, show significant mortality benefit compared to previous research [3–7]. However, many patients still die from sepsis around the world each year, with international care standards rarely achieved in full [8–10]. One strategy adopted to improve this is using a simplified care bundle, the ‘Sepsis 6’ (Table 1), which demonstrates increased compliance and an association with reduced mortality compared to full Surviving Sepsis Campaign care bundles [11].

**Table 1:** Sepsis Six care bundle [9]

Within the first hour of recognition of sepsis:	
- Measured lactate/hemoglobin - Urine output - Blood cultures - Antibiotics - Oxygen - Intravenous fluids	

Performing the Sepsis Six requires a range of behaviors to be performed by multiple individuals at different organizational levels (e.g. nurse identifies unwell patient, junior doctor diagnoses sepsis, prescribes bundle, escalates patient to consultant and performs blood cultures, nurse administers oxygen, fluids and antibiotics.). Previous research identifies it as a complex ‘trajectory of workflow’, requiring prioritization and coordination, prone to operational failure [12]. A range of cultural, contextual and behavioral determinants are likely to influence implementation and result in variation in practice within and across hospitals. It is thus critical that quality improvement initiatives consider and address the broad spectrum of potential influences on implementation [13, 14]. However, systematic reviews identify that quality improvement initiatives in both emergency medicine and antimicrobial stewardship often fail to consider socio-behavioral factors influencing clinical decision making and practice [15, 16].

Clinical practice is a form of human behavior, and may thus be understood using theory and tools from behavioral sciences [17]. Theory provides a replicable, generalizable framework through which to understand determinants of behavior [18]. The complexity and variety of available behavioral theories has posed a barrier to their use by non-specialists [19]. The Theoretical Domains Framework (TDF) synthesizes key constructs from 33 theories relevant to healthcare professional behavior change into 14 theoretical ‘domains,’ representing the range of possible behavioral determinants, from ‘knowledge’ to ‘social influences’ and ‘environmental context and resources’ [18, 20, 21]. The TDF has been applied across a range of clinical contexts (e.g. antibiotic prescribing, transfusion) to systematically identify barriers/facilitators to healthcare professional behavior change (i.e. to conduct a ‘behavioral diagnosis’ to identify ‘what’ needs to change) [22–24]. To inform subsequent intervention design, TDF Domains have also been mapped to individual Behavioral Change Techniques (BCTs), enabling selection of BCTs that are likely to target identified barriers/facilitators [25, 26].

Recent work has used the TDF to both analyze and refine a pre-existing quality improvement intervention around Sepsis Six implementation in a single hospital. This identified several themes regarding barriers/facilitators to Sepsis Six performance: ‘Knowing what to do and why,’ ‘risks/benefits,’ ‘working together,’ ‘empowerment and support,’ and ‘staffing levels’ [27, 28]. Ethnographic work identifies the need for a systematic theory-based approach towards analyzing the complex nature of this process, for example differences in barriers and facilitators between roles or clinical areas [12]. The increasing economic squeeze on healthcare, and the large resource demands of a critically ill patient, dictate not only that basic early interventions such as the Sepsis Six are performed well, but that quality improvement is focused, effective and efficient. There have been a limited number of studies adopting a behavioral and social science approach to understanding implementation issues in sepsis care, and there is a need for further studies to explore generalizability and contribute to the limited body of knowledge in this area.

Our study therefore aims to apply the TDF to systematically identify key barriers and facilitators towards Sepsis Six performance, and conduct a ‘behavioral diagnosis’, prior to intervention development, at a different single hospital. More broadly, the study aims to demonstrate a ‘worked example’ of a replicable method for using behavioral theory in quality improvement processes within emergency medicine, and to enable systematic analysis of where problems lie for different roles and clinical areas.

## Methods

### Design and setting

Mixed-methods, two-phased study: 1) Semi-structured interviews based on the TDF, conducted with a sub-sample of relevant healthcare professionals to identify key barriers/facilitators to Sepsis Six performance; and 2) Questionnaires to explore generalizability and importance of identified barriers/facilitators.

### Setting

Acute areas of a 760-bed district general hospital in England: Emergency Department (ED), adult medical admissions (MAU) and adult surgical admissions units (SAU). Unpublished local audits of Sepsis Six performance highlight low compliance in this hospital (0–20% septic patients receiving all bundle components within one hour) [29].

### Ethical approval

Local Research and Development department approval obtained confirming Service Development Project status, therefore not requiring formal ethics committee review.

### Semi-structured interviews

#### Participants

In order to investigate barriers/facilitators from the perspective of the range of clinical staff involved in Sepsis Six implementation, participants were purposively sampled from relevant stakeholders groups, including: registered nurses, junior-level doctors, or consultant-level doctors working in ED, MAU or SAU.

Potentially eligible participants were identified by the lead investigators (NR,GH), both doctors working on the critical care unit at the study hospital. Potential participants were approached over a 6-week timeframe, either in person or by e-mail. In line with previous studies using the TDF, a minimum initial sample of ten participants was proposed for full data analysis. An eleventh participant was then analyzed, and if new beliefs emerged, sampling continued until data saturation was achieved (i.e. no new themes identified) [24, 30]. Thirteen participants were interviewed initially, with a stratified sample of one consultant, junior doctor and nurse each from ED, MAU and SAU selected as part of the initial ten analyzed transcripts, with further participants selected at random.

#### Materials

A topic guide consisting of 29 questions to elicit beliefs about Sepsis Six performance was designed based on the TDF. It included at least one question relating to each domain. Table 2 lists sample questions from this study for each domain. The topic guide was developed by 3 critical care physicians with expertise in sepsis and a health psychologist with TDF experience. The questionnaire was piloted with nurses, junior doctors and consultants from other clinical areas within the study hospital. The topic guide was revised to clarify wording and is available as Additional file 1.

**Table 2:** Theoretical domains framework with sample questions [20]

Domain	Content	Sample question as applied to this study
Knowledge	An awareness of something	What do you understand by the Sepsis Six?
Skills	Ability or proficiency acquired through practice	Can you think of any ways in which your own skills for performing the steps in the Sepsis Six could be improved?
Social/professional role and identity	Set of behaviors and qualities of an individual in social or work setting	To what extent do you consider performing the steps in the Sepsis Six a part of your role?
Beliefs about capabilities	Views about one’s ability/talent/capability to perform the target behavior(s)	Are there any particular steps that you are more or less confident about performing?
Optimism	Confidence that things will happen for the best or that desired goals will be attained	How optimistic or pessimistic are you that improving performance of the Sepsis Six holds the potential to improve patient care in the future?
Beliefs about consequences	Acceptance of the truth, reality or validity about outcomes of a behavior in a given situation	To what extent do you believe that performing the steps in the Sepsis Six can affect patient outcomes?
Reinforcement	Increasing the likelihood of a behavior being performed by establishing an association between performing a behavior and a given stimulus or cue	Are you aware of any ways in which performing the Sepsis Six is rewarded?
Intentions	Conscious decision to perform a behavior or resolve to act in a certain way	To what extent do you intend to (continue to) perform the Sepsis Six in daily clinical practice?
Motivation and Goals	Mental representation of outcomes or states that an individual wants to achieve	Do you have any specific goals for performing the Sepsis Six*?*
Memory, attention and decision processes	The ability to retain information, focus selectively on aspects of the environment and choose between two or more alternatives	How easy or difficult is it to remember the steps involved in the Sepsis Six when you are performing it in daily clinical practice?
Environmental context and resources	Circumstances of a person’s situation/environment that affect behavior	To what extent does your working environment have sufficient levels of resources needed to allow performance of the Sepsis Six within one hour of recognition?
Social influences	Interpersonal processes that can cause individuals to change thoughts/feelings/behaviors	Are there any conflicting beliefs amongst your colleagues about the Sepsis Six?
Emotions	Complex reaction pattern by which individual attempts to deal with a personally significant matter or event	To what extent do you feel that your emotional state affects your performance of the Sepsis Six?
Behavioral regulation	Anything aimed at managing or changing objectively observed or measured actions	Do you ever receive feedback on your performance of the Sepsis Six on septic patients?

#### Procedure

Participants were invited either in person or by email. Written consent was obtained after a briefing regarding the study’s purpose. Interviews took place either in person in a private location, or by telephone, by a trained interviewer (NR, GH), and were digitally recorded. Recordings were transcribed verbatim and anonymized.

#### Analysis

Interviews were coded and analyzed in 3 discrete steps, using content analysis following a combined framework analysis approach. These are standard methods from other TDF-based studies using semi-structured interviews [31–36].

##### Pilot coding

A coding framework was developed to promote consistent coding of utterances into appropriate domains. This was adapted from previous Sepsis Six TDF research [27, 28]. To promote greater coding consistency, a pilot interview was transcribed and coded jointly by two members of the research team (NR,GH) [36]. Discrepancies were resolved through discussion.

##### Coding of participant responses into TDF domains

Participant responses were split into individual ‘utterances’ and coded into to the TDF domain it was judged to best represent. For example, *“[The Sepsis Six is] a package of care which has been shown to improve mortality in patients with sepsis.”* was allocated to “Knowledge”. Utterances corresponding to more than one domain were allocated as such, for example *“We've got really good nursing staff who can do bloods and blood cultures as well.”* was allocated to both ‘Skills’ and ‘Social and Professional Role’.

##### Thematic synthesis and generation of belief statements

Utterances within domains were compared across transcripts, and those expressing similar views were grouped together. Belief statements were then generated summarizing each group of similar utterances. Belief statements are defined as ‘a statement that provides detail about the role of the domain in influencing behavior’ [36]. For example *“It’s easy [to remember the steps]. It’s three in and three out.”* and *“Relatively easy [to remember] actually. The fact I can remember them for this interview has proven to me that they’re relatively easy to remember.”* were represented in the belief statement ‘It’s easy/difficult to remember the six steps in clinical practice’. Thematic synthesis was conducted by the lead researchers (NR,GH). Each generated belief statement was independently reviewed by a health psychologist (FL) to promote robust and defensible coding according to the TDF, and ensure the generated belief statement provided a valid representation of the constituent utterances [37]. Regular consensus discussions were held to resolve disagreement [31].

### Questionnaire

#### Participants

All staff currently eligible from the stakeholder staff groups (consultants, junior doctors and nurses from MAU, SAU and ED) were identified. Staff on long-term sick or maternity leave were excluded from the distribution list.

#### Materials

A questionnaire containing 54 two-part questions was synthesized using belief statements identified in the interviews. Belief statements concerning importance, or different perspectives on the same topic which would be answered through individual questionnaire responses (eg *“my colleagues do not have the skills.../I do not have the skills...”* were collapsed and combined. Practical usefulness of the resultant data was also factored in, for example a general ‘resource availability’ belief statement was separated into questions on ‘staff’ or ‘equipment’ in order to focus on which resources were most in need of intervention. After entering basic demographic information, participants were asked to rank, on a 5-point Likert scale, agreement with two opposing statements constructed from each belief statement. For example, *“performing the Sepsis Six IS part of my role”* scoring 1 and *“performing the Sepsis Six is NOT part of my role”* scoring 5. They were then asked to rate this statement in terms of importance to their delivery of the Sepsis Six, ranging from 1 *“very unimportant”* to 5 *“very important”.* Each domain had at least one questionnaire item associated with it. The questionnaire was available in paper format or electronically using SurveyMonkey. It was piloted with nursing and medical staff from other hospital areas (so not included in the study population), and revised to simplify formatting and wording of the Likert scale. A final version of the questionnaire is available in Additional file 2.

#### Procedure

Data collection took place over six weeks from June–July 2016. Participants were sent an invitation email. Consultants and lead nurses were asked to promote the questionnaire to staff. Paper questionnaires were accessible in clinical areas to further optimize response rate from those who may not check email. Potential participants were followed up by weekly emails, and reminders in person.

#### Analysis

Analysis was performed on Microsoft Excel. Questions were reverse scored if necessary such that all facilitator statements were associated with positive agreement scores, and all barriers associated with negative agreement scores. A median score (and interquartile range) was calculated for each part (agreement/importance) of the 54 questions, for each of 16 analysis groups, as follows: 1) overall median for the hospital (as a whole), median for 2) each role, 3) each clinical area and 4) each role *within* each clinical area.

Likert scales produce ordinal data, with no guarantee that each participant has the same baseline, or the same intervals between descriptors. Therefore, median agreement scores and importance scores *for the questionnaire as a whole* were then calculated overall for each of these 16 analysis groups, in order to establish a ‘baseline’ level of agreement and importance for each group.

#### Selection of important belief statements

Agreement and importance were assessed separately for each question. Therefore, selection of important barriers required dichotomizing the answers to both of these aspects. For each analysis group, median agreement and importance scores for each question were compared to the group’s overall questionnaire baseline agreement and importance scores. Statements were then dichotomized, being classified as barriers for an analysis group if the median agreement score for the individual question was less than the group’s baseline agreement score for the questionnaire; otherwise they were classified as facilitators. Statements were classified as ‘important’ for an analysis group if the median importance score for the individual question was equal to or above the group’s baseline importance score for the questionnaire; otherwise they were classified as ‘unimportant’. This approach allowed identification of relatively important, or relatively unimportant, barriers or facilitators, for each analysis group.

Once important barriers and facilitators were identified for each analysis group, they were compared to look for areas of ‘discord’ between roles or clinical areas, where an important barrier for some (for example, ‘Nurses’ or ‘Surgery’) was an important facilitator for others (for example, ‘Junior doctors’ or ‘Emergency Department’). Identification of discordant beliefs allows for further tailoring of subsequent quality improvement interventions.

## Results

### Semi-structured interviews

#### Participant characteristics

No new belief statements were identified after analysis of the initial ten participant interviews; therefore, thematic data saturation was deemed achieved and no further interviews were conducted or analyzed (Data saturation table in Additional file 3). Interviews lasted a mean 35.8 min (range 18–48). 70% participants were male. Three participants were consultants, three were junior doctors and four were nurses. Three participants came from MAU, three from SAU and four from ED. Participants had worked at the study hospital for a mean 6.5 years (range 0.8–17).

#### Coding of responses into TDF domains

In total, 1699 utterances were coded into 14 TDF domains.

Extracted utterances were synthesized into 64 belief statements. These are presented in Table 3 with example quotes. The most populated domains were Social and Professional role (9 belief statements) and Intentions (8 belief statements).

**Table 3:** Results of interview analysis

Domain	Belief Statement	Example Utterance	Frequency (number of interviews)
Knowledge	I know/do not know what the Sepsis Six involves	[The Sepsis Six is] a package of care which has been shown to improve mortality in patients with sepsis. (Consultant 1)	10
	My colleagues do/do not know what the Sepsis Six involves	the more senior sort of colleagues weren’t familiar with systemic inflammatory response syndrome, recognise all the sort of markers. (Nurse 4)	10
	I am aware/not aware of the evidence behind the Sepsis Six	There have been obviously clinical trials which I can’t remember the names of. (Junior doctor 2)	10
	My colleagues are aware/not aware of the evidence behind the Sepsis Six	If I’m honest then no. [My colleagues and I are not aware of the evidence behind the Sepsis Six] (Nurse 3)	2
	People would give better Sepsis Six performance if they were more aware of the later complications of poorly managed sepsis	I think if they had perhaps more awareness about how, like, poor sepsis management could affect a patient long term, they might be, they might have more urgency in carrying it all, sort of out. About what would happen in the long term. (Nurse 4)	1
	Having knowledge and understanding of the Sepsis Six does/does not influence the likelihood of it being performed	And it’s always, [giving antibiotics] tends to get done I think, because I think everyone understands the urgency. (Junior doctor 1)	9
Skills	I do/do not have the skills to perform the Sepsis Six	I mean I haven’t given antibiotics myself. (Junior doctor 1)	9
	My colleagues do/do not have the skills to perform the Sepsis Six	maybe if [nurses are] newly qualified, not being able to give the IV antibiotics because they would then have, because they haven’t done their IV pack (Nurse 1)	9
	There is/is insufficient provision of training and assessment in the skills required to perform the Sepsis Six	I think we’ve recognised that, and we’ve trained our nurses to deliver antibiotics, fluids, take blood cultures and lactates, put in urinary catheters, so we know our nurses can do all of this, we train them to do all of this. (Consultant 1)	9
Memory, Attention and Decisions	It’s easy/difficult to remember the 6 steps in clinical practice	Give 3, take 3 away. And that we have it written down on our proformas. (Consultant 1)	10
	The decision to start the Sepsis Six is not made because sepsis is not recognised	So I think I’ve got a reasonable understanding of recognising sepsis. (Junior doctor 3)	8
	Regular use of the Sepsis Six makes it easier to remember the steps	I mean when doing on a daily basis pretty easy to remember. But I guess if you’re not doing it on a daily basis you might forget (Junior doctor 2)	7
Behavioral Regulation	Sepsis Six performance is (not) monitored or audited regularly in my department	[Sepsis 6 is audited on a] weekly basis and the results are published weekly. (Junior doctor 3)	10
	I/we get insufficient feedback on our Sepsis Six performance	Yes it would be helpful to have more monitoring systems in place. And individual feedback to clinicians. (Consultant 2)	9
	There are sufficient tools in place to help guide and track Sepsis Six performance in individual patients	Yes, we’ve got [a Sepsis 6 tool], it’s at the back of the pro forma and the BUFALO stickers. So there’s a lot of guidance. (Consultant 3)	10
	Improving sepsis care and Sepsis Six performance is (not) discussed in regular meetings in my department	What we’re doing in surgery is auditing this sort of thing on a monthly basis, and that’s going to be presented at governance meetings. (Consultant 2)	5
	Sepsis Six performance improves if we are involved in the quality improvement process	Yeah, I think so, because I think people would own things more if they felt it was, they were included in it (Nurse 2)	3
	There are (no) action plans to improve Sepsis Six performance	there are other things that we’re doing such as implementing junior doctor training, nurse training on sepsis, through educational sessions, through induction. (Consultant 1)	4
Social Influences	My colleagues opinions do/do not affect my performance of the Sepsis Six	I don’t think the opinions of my colleagues does affect whether I do the Sepsis Six actually. (Junior doctor 3)	10
	My Colleagues do/do not believe that the Sepsis Six is beneficial to patient care	Yeah I think it’s generally believed that these steps benefit patient outcomes, so I think everyone’s kind of in favour of them. (Junior doctor 2)	9
	Departmental culture facilitates/hinders performance of the Sepsis Six	I think that’s because there isn’t a culture of doing fluid charts on every patient that comes through us (Consultant 1)	4
	There is insufficient leadership to improve Sepsis Six performance	But if there was a clear strategy, a clearer kind of team role, leadership role for the patients, the benefits of it, can’t see why it wouldn’t be used and why it couldn’t improve. (Consultant 3)	7
	Healthcare workers do/do not feel able to escalate up the hierarchy	if you have it on a care pathway, that gives them allowance, permission almost to phone the consultant and escalate it, so they’re allowed to do that, rather than feeling I shouldn’t do this. (Consultant 2)	4
	Having a Sepsis “Champion” would/would not improve performance of the Sepsis Six	it might be beneficial if, other wards as well to have a designated sepsis champion or link nurse as such, so that we can perhaps hold regular meetings every couple of months, to see how we can make changes to sepsis care. (Nurse 4)	3
Social and Professional Role	Performing the steps in the Sepsis Six is (not) my role	I think they’re all part of it. (Consultant 2)	10
	Performing all steps in the Sepsis Six is (not) my colleagues’ role	I think it should be everyone’s responsibility and role to do it. (Consultant 3)	3
	It is my/my colleagues’ role (doctor/nurse/HCA) to identify septic patients	our nurses are very good at identifying sick patients. (Junior doctor 3)	5
	It is my role to decide when to perform the Sepsis Six	we usually don’t give oxygen to somebody unless their sats are low, but in this instance, occasionally I’ve been told by a surgeon, I want them to have 2 l of oxygen. (Nurse 2)	4
	There is high turnover of medical/nursing staff in areas looking after septic patients	Our medical staff, so half of them are transient, half of them are permanent. (Consultant 1)	3
	My role is to improve Sepsis Six performance through non-clinical factors (leadership, support, supervision)	My role is that even if the patient is stable to ensure all the steps had been followed, and to reinforce and educate. (Consultant 3)	4
	There are some steps in the Sepsis Six which I/my colleagues do not/are not allowed to perform	I don’t know, as a trust I don’t think the nurses usually take blood cultures, it seems to be a doctor role. (Nurse 2)	6
	Non-clinical staff (eg bed management) put pressure on clinical staff to prioritise tasks other than Sepsis Six	And there’s such a drive for discharging them, and getting patients out, and often the bed manager puts so much pressure on the nursing staff on the wards. (Consultant 2)	1
	Staff should be empowered to improve their role in Sepsis Six performance	So I think being involved in the audit kind of made us kind of aware of what needs to be done. (Junior doctor 3)	2
Environment, Context and Resources	I do (not) have sufficient resources (staff; time; equipment; medicines; bed) to perform the Sepsis Six in one hour.	Not enough beds (Consultant 2)	10
	The equipment I have does/doesn’t work	our gas machine is down a lot of the time (Junior doctor 3)	8
	The layout of the hospital hinders/helps my performance of the Sepsis six in one hour (patient location, equipment, medicine).	trying to get a patient seen and then treated within that time, and then if they’re coming up to 4 h of being in the department are they moved to another ward before their treatment sort of is delivered, (Nurse 4)	8
Belief in Consequences	Performing the steps in the Sepsis Six improves patient outcomes	Yeah, I believe it’s vital. There’s evidence out there which supports, supports it, so, yeah (Consultant 2)	10
	The benefits of performing the Sepsis Six outweigh the risks	I think generally the advantages should outweigh the risks. (Junior doctor 2)	8
	The benefits vs risks of performing the Sepsis Six (or some parts of it) are (not) different in certain patient groups	I think any patient with known heart problems, I’d be a little bit more careful. (Nurse 3)	10
	The quicker the steps can be delivered, the more impact they have	Well, I believe if it’s carried out promptly within the 1 h then it can definitely improve the patient’s outcome. (Nurse 3)	5
	Early and regular reassessment of patients requiring the Sepsis Six gives the best outcomes	I think if you keep doing them without reassessment then that would lead to problems, but in the first hour I don’t think it’s an issue. (Consultant 2)	1
Belief in Capabilties	I am (not) confident performing the steps in the Sepsis Six	I think, you know, I’ve got the skill to perform these 6 steps, there’s no doubt about it. The training has been there, I have the skill to do it (Consultant 1)	9
	My colleagues are (not) confident performing the steps in the Sepsis Six	I mean I would hope most people were... but yeah, I think most people are confident that I’ve seen (Junior doctor 2)	7
	Some of the Sepsis Six steps are more difficult than others to achieve (urine output, cultures, antibiotics)	it’s just the urine output measurement which causes ongoing difficulties, (Consultant 1)	9
	There is good/poor communication and teamwork between members of the team looking after septic patients	It’s definitely a team priority to be able to carry it all out, so if we can sort of work together, I believe that it can be done a lot quicker, rather than doing it single-handedly. (Nurse 4)	10
	We provide good sepsis care at this hospital	I think for the most part our septic patients is reasonably well recognised via the acute care bundle, because they come in, they have the acute care pathway, filled out for every patient (Consultant 2)	1
	I am confident looking after sick septic patients	I’m very good at dealing with a crisis and just getting on with it (Nurse 2)	1
Intentions	I (don’t) prioritise performing the Sepsis Six on a septic patient over other tasks	I think unless somebody was having a cardiac arrest I would prioritise this probably above most other things. (Nurse 2)	10
	I intend to improve my knowledge of the Sepsis Six	I think I do need to know a bit more about it, so I might try and educate myself before I go back to work (Nurse 2)	2
	I intend to continue to perform the Sepsis Six on septic patients	I guess, well I’ll carry on carrying it out until it’s, unless there’s anything else new that comes up that improves sepsis care (Nurse 4)	8
	I am more likely to complete all steps of the Sepsis Six if I think the patient is sick/less likely if they are well	if we are concerned someone really is poorly, then they often will become catheterised (Nurse 2)	5
	Sometimes I choose (not) to complete the full Sepsis Six because the risks and benefits are different for that patient/situation.	your octogenarian who’s got sepsis, you might not go chucking in 2 l immediately. (Consultant 2)	7
	My colleagues (don’t) prioritise performing the Sepsis Six on a septic patients over other tasks	but it’s usually the other pressures that we have on, like prioritising other patients for example, and how big our caseload is at that time (Nurse 4)	2
	Some steps in the Sepsis Six are more/less important than others	I like having the fluids here quickly. That’s one of the better ones, I think (Nurse 3)	6
	I (don’t) perform the Sepsis Six despite not having a confirmed diagnosis because I (don’t) believe the risks of undertreating sepsis outweigh the risks of performing the Sepsis Six	they’re not septic, but they just got a big SIRS response and they looked septic when they came in, so having antibiotics in that situation is not the wrong thing to do, as a one off, but a patient’s presenting with peritonitis or what’s not, they need to have early sepsis source control. (Consultant 2)	3
Goals	I work towards a goal that the Sepsis Six should be completed and documented within an hour on all septic patients.	We should do it on all, it should be done within an hour. (Consultant 3)	10
	The hospital has/does not have a goal of improving Sepsis Six compliance	I know that the Trust is starting a BUFALO[sic] to help people remember how to deliver the Sepsis Six (Nurse 4)	10
Optimism	Sepsis Six compliance at this hospital will (not) improve	Knowing how well in general all the care bundles are used, unless there’s a clear strategy on how to improve it, my worry would be that it might not improve significantly (Consultant 3)	4
	Increasing Sepsis Six compliance will improve patient care	I’m very optimistic that if we can push this forward that it will hugely improve patient care, and their outcome. (Nurse 2)	9
Reinforcement	Individuals are not formally rewarded or punished for (failing to) complete the Sepsis Six	Not punished, obviously it’s audited, and the departments are fed back (Junior doctor 2)	10
	The department or hospital is (not) formally rewarded or punished for (failing to) complete the Sepsis Six	No, I’m not aware of any ways in which we as, do you mean as a trust are punished? (Nurse 4)	6
Emotions	I get emotionally affected negatively/positively by managing septic patients	I mean obviously you do [get affected emotionally by looking after septic patients], if they’re unwell (Junior doctor 2)	9
	If we are affected emotionally (eg stressed, excited, fatigued) it leads to better/worse clinical performance when looking after septic patients	Well if anything it makes me go, try and make, do it faster because I recognise that they’re quite sick. (Junior doctor 1)	9
	I feel good if I deliver the Sepsis Six/bad if I don’t deliver the Sepsis Six to a septic patient	I try to carry it out within the 1 h, and when it hasn’t happened I kind of feel, like frustrated (Nurse 4)	4

### Questionnaire

#### Participant characteristics

Two hundred fifty-five potential participants were invited to complete the questionnaire. 54 of these were consultants (36 medical, 10 surgical, 8 ED), 82 junior doctors (32 medical, 27 surgical, 23 ED), and the remaining 119 were nurses (38 medical, 18 surgical, 63 ED). Response rates are given in Table 4.

**Table 4:** Survey response rates

Area and Role	Number invited	Number completed	Response rate
MAU Consultants	36	15	41.7%
MAU Junior Doctors	32	15	46.9%
MAU Nurses	38	14	36.8%
SAU Consultants	10	7	70.0%
SAU Junior Doctors	27	12	44.4%
SAU Nurses	18	12	66.7%
ED Consultants	9	7	77.8%
ED Junior Doctors	22	7	31.8%
ED Nurses	63	24	38.1%
Total	255	113	44.3%

Length of time participants had worked in their current role ranged from 4 months to 30 years.

#### Belief statements

Forty-six belief statements were ‘important facilitators’ for at least one analyzed participant group. 30 belief statements were ‘important barriers’ for least one analyzed participant group.

Status of each belief statement amongst the overall sample is presented in Table 5, grouped by domain. Discordance is illustrated here by the number of groups for whom a belief statement was an important barrier, against those for whom it was an important facilitator. The complete results table, with median agreement and importance scores and interquartile ranges for each participant group for each belief statement, is presented in Additional file 4. Questionnaire results are discussed by domain in the following text.

**Table 5:** Classification of belief statements according to questionnaire results, presented by domain

Domain	Barrier belief	Facilitator belief	Overall sample group	Important barrier (number of analysis groups)	Unimportant barrier (number of analysis groups)	Unimportant facilitator (number of analysis groups)	Important facilitator(number of analysis groups)
Belief in Capabilities	There is POOR teamwork when looking after septic patients	There is GOOD teamwork when looking after septic patients	Important barrier	10	0	0	6
	I am NOT confident performing the Sepsis Six	I AM confident performing the Sepsis Six	Important enabler	0	0	0	16
	Some of the steps in the Sepsis Six are MORE DIFFICULT to perform than others	The steps in the Sepsis Six are EQUALLY EASY OR DIFFICULT to perform	Unimportant barrier	9	4	0	3
	There is POOR communication between members of the team looking after septic patients	There is GOOD communication between members of the team looking after septic patients	Important barrier	11	0	0	5
	We provide POOR sepsis care at this hospital	We provide GOOD sepsis care at this hospital	Unimportant barrier	8	3	0	5
Belief in Consequences	Delivering the Sepsis Six quickly does NOT increase how much benefit it has	Delivering the Sepsis Six quickly DOES increase the benefit it has	Important enabler	0	0	0	16
	Performing the steps in the Sepsis Six does NOT improve patient outcomes	Performing the steps in the Sepsis Six DOES improve patient outcomes	Important enabler	0	0	0	16
	Overall, the RISKS of performing the Sepsis Six outweigh the benefits	Overall, the BENEFITS of performing the Sepsis Six outweigh the risks	Important enabler	1	0	0	15
	The RISKS of performing the Sepsis Six outweigh the benefits in CERTAIN patient groups	The BENEFITS of performing the Sepsis Six outweigh the risks in ALL patient groups	Unimportant barrier	6	3	1	6
	Early and regular reassessment of patients requiring the Sepsis Six has NO effect on outcomes	Early and regular reassessment of patients requiring the Sepsis Six gives the BEST outcomes	Important enabler	0	0	0	16
Behavioral Regulation	Sepsis Six performance is NOT audited regularly in my department	Sepsis Six performance IS audited regularly in my department	Unimportant barrier	2	5	4	5
	There are INSUFFICIENT tools in use to guide & track Sepsis Six performance in individual patients	There are SUFFICIENT tools in use to guide & track Sepsis Six performance in individual patients	Unimportant barrier	5	4	2	5
	We get INSUFFICIENT feedback on our Sepsis Six performance	We get SUFFICIENT feedback on our Sepsis Six performance	Unimportant barrier	8	7	0	1
	Sepsis Six performance is NOT discussed in meetings in my department	Sepsis Six performance IS discussed in meetings in my department	Unimportant barrier	4	2	4	6
	Involving clinical staff in Sepsis Six performance improvement will NOT lead to greater improvement	Involving clinical staff in Sepsis Six performance improvement WILL lead to greater improvements	Important enabler	0	0	1	15
	There are NO plans in place to improve Sepsis Six performance at my hospital	There ARE plans in place to improve Sepsis Six performance at my hospital	Important barrier	2	1	2	11
Environment, Context and Resources	There is INSUFFICIENT staffing to perform the Sepsis Six	There is SUFFICIENT staffing to perform the Sepsis Six	Important barrier	13	0	0	3
	There is INSUFFICIENT time to perform the Sepsis Six	There is SUFFICIENT time to perform the Sepsis Six	Important barrier	11	0	0	5
	There is INSUFFICIENT equipment / medication to perform the Sepsis Six	There is SUFFICIENT equipment / medication to perform the Sepsis Six	Important barrier	5	0	0	11
	There are INSUFFICIENT beds available in my department to look after septic patients	There are SUFFICIENT beds available in my department to look after septic patients	Important barrier	15	0	0	1
	The equipment I need to perform the Sepsis Six does NOT work or works poorly	The equipment I need to perform the Sepsis Six DOES work well	Important enabler	3	0	0	13
	Septic patients are RARELY managed in an appropriate location	Septic patients are ALWAYS managed in an appropriate location	Important barrier	15	0	0	1
Emotions	I do NOT feel bad if I do not deliver the Sepsis Six to a septic patient	I DO feel bad if I do not deliver the Sepsis Six to a septic patient	Important enabler	0	0	1	15
	I do NOT feel anxious/stressed when treating septic patients	I DO feel anxious/stressed when treating septic patients	Unimportant barrier	3	13	0	0
Goals	I do NOT have a time-based goal for completing the Sepsis Six on septic patients	My goal is to complete the Sepsis Six within an HOUR on all septic patients	Important enabler	0	0	0	16
Intentions	I do NOT intend to improve my knowledge of the Sepsis Six	I INTEND to improve my knowledge of the Sepsis Six	Unimportant barrier	1	6	2	7
	When uncertain about diagnosis I WAIT FOR CONFIRMATION of sepsis before performing the Sepsis Six	When uncertain about diagnosis I PERFORM the Sepsis Six rather than miss treating potential sepsis	Important enabler	2	1	0	13
	I do NOT intend to continue to perform the Sepsis Six on septic patients	I DO intend to continue to perform the Sepsis Six on septic patients	Important enabler	0	0	0	16
	I am UNLIKELY to complete all steps of the Sepsis Six if I think the patient is well	I am LIKELY to complete all steps of the Sepsis Six even if I think the patient is well	Unimportant barrier	7	8	1	0
	I do NOT prioritise performing the Sepsis Six on a septic patient over other tasks	I DO prioritise performing the Sepsis Six on a septic patient over other tasks	Important enabler	2	0	1	13
	SOME steps in the Sepsis Six are more or less important than others	ALL steps in the Sepsis Six are equally important	Unimportant barrier	6	4	1	5
Knowledge	I am NOT aware of what the Sepsis Six involves	I AM aware of what the Sepsis Six involves	Important enabler	0	0	0	16
	I am NOT aware of the evidence supporting the Sepsis Six	I AM aware of the evidence supporting the Sepsis Six	Unimportant enabler	0	1	2	13
Memory, Attention and Decisions	It’s DIFFICULT to remember all the steps of the Sepsis Six in day-to-day clinical practice	It’s EASY to remember all the steps of the Sepsis Six in day-to-day clinical practice	Important enabler	0	0	0	16
	I OFTEN miss sepsis	I RARELY miss sepsis	Important barrier	7	0	0	9
	Regular use of the Sepsis Six does NOT make it easier to remember the steps involved	Regular use of the Sepsis Six DOES make it easier to remember the steps involved	Important enabler	0	0	3	13
Optimism	Sepsis Six performance at this hospital will NOT improve	Sepsis Six performance this this hospital WILL improve	Important barrier	3	1	0	12
	Increasing Sepsis Six performance will NOT improve patient care	Increasing Sepsis Six performance WILL improve patient care	Important enabler	0	0	0	16
Reinforcement	The hospital is NOT formally rewarded for good Sepsis Six performance	The hospital IS formally rewarded for good Sepsis Six performance	Unimportant barrier	0	15	1	0
	Individuals are NOT formally rewarded for good Sepsis Six performance	Individuals ARE formally rewarded for good Sepsis Six performance	Unimportant barrier	2	14	0	0
Social Influences	The culture within my department HINDERS performance of the Sepsis Six	The culture within my department HELPS performance of the Sepsis Six	Important barrier	6	0	0	10
	There is INSUFFICIENT leadership for improving Sepsis Six performance	There is SUFFICIENT leadership for improving Sepsis Six performance	Unimportant barrier	6	1	1	8
	My colleagues’ opinions about the Sepsis Six do NOT affect whether I perform it	My colleagues’ opinions about the Sepsis Six DO affect whether I perform it	Unimportant barrier	5	10	0	1
	My colleagues do NOT believe that the Sepsis Six is beneficial to patients	My colleagues DO believe that the Sepsis Six is beneficial to patients	Unimportant enabler	0	0	3	13
	I do NOT feel able to escalate when I am concerned about a patient who may need the Sepsis Six	I DO feel able to escalate when I am concerned about a patient who may need the Sepsis Six	Important enabler	0	0	0	16
	Having a local sepsis ‘champion’ would NOT improve performance of the Sepsis Six	Having a local sepsis ‘champion’ WOULD improve performance of the Sepsis Six	Unimportant barrier	0	12	2	2
Skills	I do NOT have the necessary skills to perform the Sepsis Six	I HAVE the necessary skills to perform the Sepsis Six	Important enabler	0	0	1	15
	There is INSUFFICIENT provision of training required to perform the Sepsis Six	There is SUFFICIENT provision of training required to perform the Sepsis Six	Important barrier	9	0	0	7
Social and Professional Role	It is NOT part of my role to decide when to perform the Sepsis Six	It IS part of my role to decide when to perform the Sepsis Six	Important enabler	0	0	0	16
	Performing the Sepsis Six is NOT part of my role	Performing the Sepsis Six IS part of my role	Important enabler	0	0	0	16
	It is NOT part of my role to identify septic patients	It IS part of my role to identify septic patients	Important enabler	0	0	0	16
	There is RAPID turnover of medical/nursing staff in areas looking after septic patients	There is SLOW turnover of medical/nursing staff in areas looking after septic patients	Unimportant barrier	10	6	0	0
	It is NOT part of my role to improve Sepsis Six performance through leadership & support	It IS part of my role to improve Sepsis Six performance through leadership & support	Unimportant enabler	0	0	2	14
	There are some steps in the Sepsis Six which I am NOT ALLOWED to perform	I am ALLOWED to perform all steps in the Sepsis Six	Important enabler	2	1	2	11

Sixteen analysis groups in total: Overall sample (1), 3 departments (MAU, ED, Surgery), 3 roles (Consultants, nurses, junior doctors) and 9 roles within departments (e.g. ED Nurses)

### Behavioral regulation

Belief within this domain focused on audit, feedback, improvement plans and the discussion of sepsis in governance meetings. There were clear differences between departments and roles, with a trend towards beliefs in this domain being barriers for participants. Particularly the surgical department showed important barriers, along with the junior doctors of MAU. Overall there was a general perception of ‘not enough feedback’ (8 analysis groups), with the notable exception of ED nurses, for whom the detailed feedback they received was an important facilitator. The strength of belief amongst MAU juniors was sufficient to classify lack of an improvement plan as an important barrier for the overall sample (2 groups), despite the converse being an important facilitator for eleven other groups. An exception to the trend for this domain was a widespread belief that involving clinical staff in performance improvement processes would lead to greater improvements (15 groups).

### Belief in capabilities

Though all 16 groups believed their confidence in the Sepsis Six itself were an important facilitator, non-technical skills such as teamwork and communication were noted as important barriers for most groups (10/16 and 11/16 respectively). Discord was also observed with these beliefs, whereby confidence in such skills were important facilitators for groups within the surgical department, and for ED juniors (6/16 and 5/16 respectively).

### Belief in consequences

Overall, this domain included important facilitators across all 16 groups, with important beliefs in the beneficial consequences of timely Sepsis Six performance and of benefits versus risks of the bundle. Notable exceptions here are a belief amongst MAU and ED doctors that the risks of the Sepsis Six outweigh the benefits in certain patient groups (e.g. oxygen in chronic obstructive pulmonary disease or intravenous fluids in cardiac patients). This was not reflected in surgical participants, who believed the Sepsis Six was always of benefit in their patients.

### Emotions

This was a mixed domain, with 15 groups expressing regret if they failed to deliver the Sepsis Six as an important facilitator, but unimportant barrier beliefs amongst 13 groups about feeling too calm and relaxed when treating septic patients. Interview participants expressed that staff should be more worried and more stressed about sepsis, as it can be so indolent when compared to more obvious emergencies like bleeding.

### Environment, context and resources

This domain included numerous barriers, particularly for ED and MAU. Reported important barriers included insufficient staff (13 groups), time (11 groups), equipment (5 groups), and beds (15 groups) to adequately deliver the Sepsis Six. Participants from ED reported having sufficient necessary equipment, however, encountered difficulties using it (e.g. gas machine in ED resus is often faulty). In contrast, equipment on MAU worked well but there were often insufficient levels (e.g. lack of drip stands for fluids). There were also concerns regarding location of care (15 groups), for example with surgically septic patients coming in via ED and MAU.

### Goals

A time-based goal of completing the Sepsis Six care bundle within one hour was an important facilitator endorsed by all 16 groups.

### Intentions

Facilitators within this domain included prioritization of septic patients (13 groups), intending to continue performing the bundle (16 groups), performing it if uncertain rather than waiting for confirmation of sepsis (13 groups), and intending to improve knowledge of the bundle (7 groups). Barriers included participants believing that some steps were more important than others (6 groups), and being unlikely to complete the full bundle if they believed the patient to be well (6 groups). Discord throughout this domain was evident in ED staff expressing strong competing priorities compared to other groups.

### Knowledge

This was an important facilitator domain – most participants knew about the Sepsis Six bundle (16 groups), and the evidence supporting it (13 groups).

### Memory, attention and decisions

Overall this domain had various facilitators towards Sepsis Six performance, with participants finding the bundle easy to remember (16 groups), and that using it regularly helps this (13 groups). There was one strongly discordant belief, with about half of participant groups reporting that missing sepsis was an important barrier for them compared to the other groups who believed that for them, rarely missing sepsis was an important facilitator.

### Optimism

All sixteen groups believed that improving performance of the bundle would improve patient care. There was a strongly discordant belief about whether performance of the bundle at the hospital would improve or not, with pessimism amongst ED and MAU juniors making this a barrier for the overall sample (3 groups), despite the optimistic facilitator belief being true for many others (12 groups).

### Reinforcement

This domain featured two barrier beliefs, with neither individuals nor the hospital being rewarded for performing the bundle. Despite this, most participants reported these barriers as unimportant barriers to their ongoing Sepsis Six performance.

### Skills

Most participants believed their skills in the Sepsis Six itself to be an important facilitator (15 groups). There was, however, a strongly discordant belief regarding whether training in the bundle was sufficient or not, with around half of the groups seeing lack of training as an important barrier (7 groups) – compared to the remaining 9 groups who believed the training they received was an important facilitator.

### Social and professional role

Overall this was a domain of important facilitators – all sixteen groups expressed as important facilitators that most aspects of Sepsis Six performance were part of their role, from identification of septic patients, deciding to perform the bundle to performing it themselves. One common important barrier was the high turnover of both medical and nursing staff in their roles (10 groups). A discordant theme was how much of the Sepsis Six a participant was actually allowed to perform. The majority of groups found this as a strong facilitator (11 groups), but medical and especially surgical nurses (2 groups) felt restricted and found this limitation of role to be an important barrier. Of note, ED nurses, who regularly do venous gases, blood cultures and have recently had protocols implemented to allow them to give the first dose of antibiotics whilst waiting for medical review, expressed this belief as a strong facilitator.

### Social influences

This domain had both important facilitators and barriers. Most participants (13 groups) expressed as a strong facilitator that they could escalate septic patients, and that they felt that their colleagues believe the Sepsis Six to be of benefit (16 groups). There were many areas of discord - departmental culture was an important barrier for six groups but a facilitator for the other ten, for example there was a culture of not measuring a urine output in ED. Six groups expressed lack of leadership in improving bundle performance as an important barrier, compared to eight groups expressing good leadership in bundle performance as an important facilitator.

## Discussion

This study illustrates a structured and replicable theory-based approach to identify multiple barriers and facilitators towards performing the Sepsis Six in admissions areas of a large UK hospital. This addresses a recently identified gap in theory-based implementation research in emergency care, and illustrates a method which could be used to improve implementation of other similar interventions [15]. There are multiple barriers, including insufficient resources, insufficient training, poor communication and teamwork and lack of audit or feedback on performance. However, participants were confident in their knowledge and skills when performing the bundle and believed it to be beneficial to their patients. Whilst some beliefs are common between participant groups, there are many areas of discord identified where beliefs that are relative barriers for one group are relative facilitators for another.

### Where does this fit with previous TDF-based research?

Previous TDF-based research into Sepsis Six performance [27, 28] identified several broad themes affecting Sepsis Six performance which this study supports: “Knowing what to do and why” is supported by this study’s facilitator beliefs around knowledge of the Sepsis Six and its supporting evidence, and facilitator beliefs around skill levels. “Risks and benefits” is supported by this study’s important facilitator beliefs in the ‘Beliefs about consequences’ domain, with widespread beliefs that the bundle is of benefit, with a few concerns about some elements in certain patient groups, for example fluids in heart failure. “Working together” was an important discordant theme identified in ‘Beliefs about capabilities’ and ‘Social Influences’ domains, and was variably an important barrier or facilitator, as were beliefs in the ‘Social Influences’ and ‘Behavioral Regulation’ domains based around “Empowerment and Support”. “Staffing levels” is a theme widely addressed in the important barrier beliefs from this study’s ‘Environment, Context and Resources’ domain. However, this study builds on previous research through identification of specific belief statements, and areas of discord between clinical areas and staff groups. This is vitally important when considering intervention design. For example, ED nurses had enough equipment like antibiotics or access to oxygen, but had problems with some of its function – such as the blood gas machine, whereas medical nurses had functional equipment but not enough of it. Being able to systematically identify detail such as this allows appropriate focus of interventions – in this case, on putting a 24-h repair service in place for the ED gas machine, and reviewing MAU equipment to allow purchase of shortage items such as drip stands.

### Where does this fit in with previous ethnographic analysis?

Previous ethnographic research in Scotland identifies a wide spectrum of complex systematic barriers and facilitators towards Sepsis Six performance, far beyond the six simple steps the bundle is intended to be. Many of these themes are also identified in the site studied here, for example the importance of teamwork and communication, problems when prioritizing competing tasks, and maintaining a purpose in completing the whole bundle when acting in a system under pressure [12].

More pertinently, ethnographic analysis of ongoing Sepsis Six quality improvement work suggested a benefit in applying systematic methods when analyzing elements of complex task performance to identify where problems lie. This is supported by the presence of discordant themes seen in this study, with some beliefs acting as barriers for one role but facilitators for another. Additionally, this study has identified the pressures brought about by the system, such as extreme pressure of resources or difficulties prioritizing, looking beyond the usual implementation approach of individual behavior change [12]. As suggested in the ethnographic analysis, this study presents a method that uses a theory-based approach that looks at both individuals and systems when analyzing barriers and facilitators to implementation.

### Strengths and limitations of using the TDF to address sepsis six implementation

The Sepsis Six is intended to be simple yet is poorly performed in day-to-day clinical practice. The systematic methods used in this paper provide an in-depth insight into factors affecting this performance in staff groups at the study hospital. Whilst many identified beliefs may have been intuitive, such as those identifying resource shortages, the TDF-based method identified a broad spectrum of contextualized beliefs beyond this, such as leadership of the Sepsis Six quality-improvement process. The frequency and importance of these beliefs are likely to differ between and across individuals and groups, and thus the use of questionnaires to assess generalizability and importance of beliefs allowed identification of which staff groups and clinical areas perceive them as barriers or facilitators. Furthermore, identification of discordant beliefs between staff groups, facilitates more targeted use of behavior change techniques and hence better-designed interventions to improve performance of clinical behaviors such as the Sepsis Six – for example, a technique to improve teamwork could be focused on MAU or ED rather than surgery given findings that poor teamwork was an important barrier for these departments but an important facilitator for surgery.

TDF domains have also been linked to Behavior Change Techniques (BCTs) in order to create a ‘treatment’ intervention for the ‘behavioral diagnosis’. Previous TDF-based Sepsis Six research has then been used to systematically guide the design of an intervention at the study hospital [26, 27]. The data collected in this study could be used to do similar.

There were several aspects of the method designed to reduce subjectivity during the analysis, including regular, iterative, multidisciplinary consensus discussions to critique the data and emerging analysis.

There may have been a degree of self-selection bias whereby those who completed the questionnaire were more likely to be engaged in quality improvement or interested in sepsis. The response rate, out of all eligible staff, is not unreasonable when compared to similar cross-sectional TDF studies and therefore the authors believe this to be a fair and representative sample, but those who did not take part may be less motivated or knowledgeable about sepsis care, biasing the results [38]. Overall, the results had a positive skew, towards the ‘facilitator’ and the ‘important’ end of the Likert scale used. This did not affect the usefulness of the results, since a belief scoring as an important weak facilitator within an analysis group, if all other beliefs were important strong facilitators, was judged to be as relevant to address in an intervention as a belief actually scoring as a barrier. Furthermore, our analysis method allows for each participant to have a different ‘baseline’ to their Likert scale, hence the positive skew should not have affected the results in terms of producing results which are practically useful in intervention design. In addition, the results of larger participant subgroups (for example, ‘Surgery’) may have been influenced by their composition (for example if made up mostly of nurses). The pre-planned subgroup analysis aims this should not compromise the results for individual staff groups. Combining quantitative and qualitative data in this way in order to identify important beliefs, has been performed previously using the TDF [22]. The method used in this study enabled identification of pragmatic and useful results through in-depth qualitative exploration of a problem then larger scale quantitative research.

### Further research

As a single hospital study, the generalizability of results may be limited. Different hospitals have different pressures and cultures as demonstrated by differences between roles and departments in this study. However, the concordance seen between this study and the previous single center and multi-center studies suggest there may be common themes in factors affecting Sepsis Six delivery between hospitals. If identified, these could be targeted for behavior change intervention at a national level. Completion of the questionnaire in other hospitals to assess for generalizability of results would systematically identify any targets for larger scale quality improvement, or reinforce the need for other hospitals to undertake a similar process through identification of discordance between participant groups. A multicenter study is currently underway using the questionnaire produced in this study.

## Conclusions

Overall, this study describes a systematic, replicable method for identifying targets for behavior change in order to improve performance of an intervention, in this case the Sepsis Six. There were common barriers relating to lack of staffing and other resources, variable audit and feedback, variable communication, leadership and teamwork amongst staff groups. However there was a good knowledge and skill-base relating to the Sepsis Six, firm belief in the positive consequences of the bundle, and strong beliefs in it being part of each participant’s role to identify septic patients and perform the bundle. Hospitals could use theory-based methods such as this to systematically identify barriers and facilitators towards their own sepsis bundle performance. They could then use these data to systematically guide design of an intervention. Conducting a behavioral diagnosis using this method could facilitate better and more efficient performance improvement not only in sepsis but throughout acute care.

## Additional files

Additional file 1: Barriers and FaciLitators to Implementing the Sepsis Six (BLISS) Topic Guide. (DOC 88 kb)

Additional file 2: Barriers and Levers to Implementing the Sepsis Six (BLISS) Questionnaire. (DOCX 67 kb)

Additional file 3: Table demonstrating belief statement occurrence and data saturation in analyzed interviews (Participants 1–10) with absence of new themes in Participant 11. (DOCX 30 kb)

Additional file 4: Complete results table with median scores and interquartile ranges (IQR). (DOCX 225 kb)

## Abbreviations

BCT: Behavior Change Technique
ED: Emergency Department
MAU: Medical Admissions Unit
SAU: Surgical Admissions Unit
TDF: Theoretical Domains Framework

## References

[1] Singer M, Deutschman C, Seymour C, Shankar-Hari M, Annane D, Bauer M (2016). The third international consensus definitions for sepsis and septic shock (Sepsis-3). JAMA.

[2] NHS England Commissioning for Quality and Innovation (CQUIN) Guidance for 2015–16 (2015) https://www.england.nhs.uk/wp-content/uploads/2015/03/9-cquin-guid-2015-16.pdf (Last accessed 26^th^ June 2016).

[3] Rivers E, Nguyen B, Havstad S (2001). Early goal-directed therapy in the treatment of severe sepsis and septic shock. N Engl J Med.

[4] Kaukonen KM, Bailey M, Suzuki S, Pilcher D, Bellomo R (2014). Mortality related to severe sepsis and septic shock among critically ill patients in Australia and New Zealand, 2000-2012. JAMA.

[5] The ProCESS Investigators (2014). A randomized trial of protocol-based care for early septic shock. N Engl J Med.

[6] The ARISE Investigators and the ANZICS Clinical Trials Group (2014). Goal-directed resuscitation for patients with early septic shock. N Engl J Med.

[7] The ProMISe Trial Investigators (2015). Trial of early, goal-directed resuscitation for septic shock. N Engl J Med.

[8] Cronshaw HL (2011). Impact of the surviving sepsis campaign on the recognition and management of severe sepsis in the emergency department: are we failing?. Emerg Med J.

[9] Kissoon N (2014). Sepsis guideline implementation: benefits, pitfalls and possible solutions. Crit Care.

[10] Kuo J, Chang H, Wu P (2012). Compliance and barriers to implementing the sepsis resuscitation bundle for patients developing septic shock in the general medical wards. J Formos Med Assoc.

[11] Daniels R, Nutbeam T, McNamara G, Galvin C (2011). The sepsis six and the severe sepsis resuscitation bundle: a prospective observational cohort study. Emerg Med J.

[12] Tarrant C, O’Donnell B, Martin G (2016). A complex endeavour: an ethnographic study of the implementation of the sepsis six clinical care bundle. Implementation Sci.

[13] Charani E (2013). E. Castro-Sanchez, N. Sevdalis, Y. Kyratsis, L. Drumright, N. Shah, and A. Holmes. "Understanding the determinants of antimicrobial prescribing within hospitals: the role of “prescribing etiquette”.". Clin Infect Dis.

[14] Davey P (2015). Time for action—improving the design and reporting of behavior change interventions for antimicrobial stewardship in hospitals: early findings from a systematic review. Int J Antimicrob Agents.

[15] Tavender EJ, Bosch M, Fiander M, Knott JC, Gruen RL, O’Connor D. Implementation research in emergency medicine: a systematic scoping review. EMJ 2015;(ePublished ahead of print). 10.1136/emermed-2015-205053 26353921

[16] Charani E, Edwards R, Sevdalis N, Alexandrou B, Sibley E, Mullett D, Franklin B (2011). Behavior change strategies to influence antimicrobial prescribing in acute care: a systematic review. Clin Infect Dis.

[17] Eccles M, Grimshaw J, Walker A (2005). Changing the behavior of healthcare professionals: the use of theory in promoting the uptake of research findings. J Clin Epidemiol.

[18] Michie S, Prestwich A (2010). Are interventions theory-based? Development of a theory coding scheme. Health Psych.

[19] Davidoff F, Dixon-Woods M, Leviton L, et al. Demystifying theory and its use in improvement. BMJ Qual Saf Published Online First: 23 January 2015. doi: 10.1136/bmjqs-2014-003627 10.1136/bmjqs-2014-003627 PMC4345989 25616279

[20] Cane J, O’Connor D, Michie S (2012). Validation of the theoretical domains framework for use in behavior change and implementation research. Implement Sci.

[21] Francis JJ, O’Connor D, Curran J (2012). Theories of behavior change synthesised into a set of theoretical groupings: introducing a thematic series on the theoretical domains framework. Implement Sci.

[22] Cuthbertson BH, Campbell MK, MacLennan G (2013). Clinical stakeholders’ opinions on the use of selective decontamination of the digestive tract in critically ill patients in intensive care units: an international Delphi study. Crit Care.

[23] Gould NJ, Lorencatto F, Stanworth SJ, Michie S, Prior ME, Glidewell L (2014). Application of theory to enhance audit and feedback interventions to increase the uptake of evidence-based transfusion practice: an intervention development protocol. Implement Sci.

[24] Roberts N, Lorencatto F, Manson J, Brundage S, Jansen J (2016). What helps or hinders the transformation from a major tertiary center to a major trauma center? Identifying barriers and enablers using the Theoretical Domains Framework. Scand J Trauma Resusc Emerge Med.

[25] Cane J, Richardson M, Johnston M (2015). From lists of behavior change techniques (BCTs) to structured hierarchies: comparison of two methods of developing a hierarchy of BCTs. British J Health Psych.

[26] Michie S, Johnston M, Francis J (2008). From theory to intervention: mapping theoretically derived behavioral determinants to behavior change techniques. Appl Psychol.

[27] Steinmo S, Fuller C, Stone S, Michie S (2015). Characterising an implementation intervention in terms of behavior change techniques and theory: the ‘sepsis six’ clinical care bundle. Implement Sci.

[28] Steinmo S, Michie S, Fuller C, Stanley S, Stapleton C, Stone S (2016). Bridging the gap between pragmatic intervention design and theory: using behavioral science tools to modify an existing quality improvement programme to implement “sepsis six”. Implement Sci.

[29] Personal communication, J Stratton, Governance Lead for Medicine, Royal Cornwall Hospital, 2015.

[30] Francis JJ, Johnston M, Robertson C (2010). What is an adequate sample size? Operationalising data saturation for theory-based interview studies. Psychol Health.

[31] McSherry LA, Dombrowski SU, Francis JJ (2012). ‘It’s a can of worms’: understanding primary care practitioners’ behaviors in relation to HPV using the theoretical domains framework. Implement Sci.

[32] Pope C, Ziebland S, Mays N (2000). Qualitative research in health care. Analysing qualitative data. BMJ.

[33] Ritchie J, Lewis J (2003). Qualitative research practice.

[34] Duncan EM, Francis JJ, Johnston M (2012). Learning curves, taking instructions, and patient safety: using a theoretical domains framework in an interview study to investigate prescribing errors among trainee doctors. Implement Sci.

[35] Curran JA, Brehaut J, Patey AM, Osmond M (2013). Understanding the Canadian adult CT head rule trial: use of the theoretical domains framework for process evaluation. Implement Sci.

[36] Islam R, Tinmouth AT, Francis JJ (2012). A cross-country comparison of intensive care physicians’ beliefs about their transfusion behavior: a qualitative study using the theoretical domains framework. Implement Sci.

[37] Bussieres AE, Patey AM, Francis JJ (2012). Identifying factors likely to influence compliance with diagnostic imaging guideline recommendations for spine disorders among chiropractors in North America: a focus group study using the theoretical domains framework. Implement Sci.

[38] Beenstock J, Sniehotta F, White M, Bell R, Milne E, Araujo-Soares V (2012). What helps and hinders midwives in engaging with pregnant women about stopping smoking? A cross-sectional survey of perceived implementation difficulties among midwives in the North East of England. Implementation Sci.

