# Supplementary material for: Barriers and facilitators towards implementing the Sepsis Six care bundle (BLISS-1): a mixed methods investigation using the theoretical domains framework
Source: Scand J Trauma Resusc Emerg Med. 2017 Sep 19;25:96. doi: 10.1186/s13049-017-0437-2 (PMC5606082; doi:10.1186/s13049-017-0437-2)
Supplement: Supplementary file 1 — Barriers and FaciLitators to Implementing the Sepsis Six (BLISS) Topic Guide. (DOC 88 kb) [file 13049_2017_437_MOESM1_ESM.doc]

**Additional file 1. Barriers and FaciLitators to Implementing the Sepsis Six (BLISS) Topic Guide**

*Opening questions:*

- What is your role at the hospital?
- How long have you worked at the hospital? And in this role?
- How often do you look after septic patients in your role – daily, weekly, monthly?

| **QUESTION NO.** | **QUESTION** | **PROMPTS** | **COMMENTS** |
| --- | --- | --- | --- |
| **KNOWLEDGE** | | | |
| 1 | What do you understand by the Sepsis Six? | - |  |
| 2 | To what extent do you know how and when to perform the steps in the Sepsis Six? | Are there any particular steps that you are more or less familiar with?  Are there any particular steps that you find your role easier or more difficult to perform?  Are there any particular steps that you are more or less confident about performing? | **note: this subprompt could also be coded as TDF domain Skills*  **note: this subprompt could also be coded as TDF domain Belief in Capabilities* |
| 3 | Are you aware of the evidence related to the Sepsis Six? |  |  |
| 4 | How familiar do you feel your colleagues are with the Sepsis Six? | Are there any particular steps that your colleagues find easier or more difficult?  Are there any particular steps that your colleagues are more or less confident in performing? | **note: this subprompt could also be coded as TDF domain Skills*  **note: this subprompt could also be coded as TDF domain Belief in Capabilities* |
| 5 | To what extent do you feel people’s knowledge about the Sepsis Six influences the likelihood of the steps being performed? |  |  |
| **SKILLS** | | | |
| 6 | Can you think of any ways in which your own skills for performing the steps in the Sepsis Six could be improved? |  |  |
| **MEMORY, ATTENTION AND DECISIONS** | | | |
| 7 | How easy or difficult is it to remember the steps involved in the Sepsis Six when you are performing it in daily clinical practice? |  |  |
| 8 | Are you aware of any systems currently in place to help remember the steps in the Sepsis Six? |  |  |
| 9 | Can you think of any ways to help people remember the steps in the Sepsis Six? |  |  |
| 10 | To what extent do other factors or priorities at work distract you from performing the Sepsis Six on a patient? |  |  |
| **ENVIRONMENT, CONTEXT AND RESOURCES** | | | |
| 11 | To what extent does your working environment have sufficient levels of resources needed to allow performance of the Sepsis Six within one hour of recognition? | *Staffing?*  *Time?*  *Equipment – amount/function?*  *Is the layout optimal? Eg Hospital/ward/treatment room*  *Any specific issues for each component of the Sepsis Six bundle (blood cultures, blood gases, oxygen, fluids, antibiotics, measurement of urine output)* |  |
| **SOCIAL AND PROFESSIONAL ROLE** | | | |
| 12 | To what extent do you consider performing the steps in the Sepsis Six a part of your role? | *Are there any steps which you feel are not part of your role?* |  |
| 13 | To what extent do you consider performing the steps in the Sepsis Six is part of the role of your colleagues (eg junior doctors/nurses/senior doctors)? |  |  |
| 14 | To what extent do you think performing the Sepsis Six within one hour of recognising a septic patient is a team or individual level behaviour? | If team – what facilitates performing the Sepsis Six as a team?  What interferes? |  |
| **SOCIAL INFLUENCES** | | | |
| 15 | To what extent do the opinions of your colleagues about the Sepsis Six affect the likelihood of you performing it? |  |  |
| 16 | Are there any conflicting beliefs amongst your colleagues about the Sepsis Six? |  |  |
| **EMOTIONS** | | | |
| 17 | To what extent do you feel that your emotional state affects your performance of the Sepsis Six? | *Any specific emotional states?* |  |
| 18 | Do you ever get affected emotionally in anyway by looking after septic patients? | *Does that impact on your performance of the Sepsis Six?* |  |
| **BELIEFS IN CONSEQUENCES** | | | |
| 19 | To what extent do you believe that performing the steps in the Sepsis Six can affect patient outcomes? | To what extent do you feel these views are shared by your colleagues? | **note: this subprompt could also be coded as TDF domain Social Influences* |
| 20 | To what extent do you think the advantages of performing the steps in the Sepsis Six outweigh the disadvantages, or vice versa? | *Are there any particular patient groups in which there are more or different advantages/disadvantages to performing these steps?* |  |
| **MOTIVATION AND GOALS** | | | |
| 21 | Compared to other tasks you have in your role, to what extent do you prioritise performing the Sepsis Six on a septic patient? |  |  |
| 22 | Do you have any specific goals for performing the Sepsis Six*?* | *Does your team?*  *What about the hospital in general?* |  |
| **REINFORCEMENT** | | | |
| 23 | Are you aware of any ways in which performing the Sepsis Six is rewarded? | *Are there any ways in which failing to complete the Sepsis Six is punished*? |  |
| **BEHAVIORAL REGULATION** | | | |
| 24 | Do you ever receive feedback on your performance of the Sepsis Six on septic patients? | *If so, from whom?*  *How often?*  *In what format?*  *Is it helpful/unhelpful?*  *How could you act on this feedback to improve practice?* |  |
| 25 | How do you and your colleagues keep track of your own performance of the Sepsis Six? | *Are you aware of any audits? If yes –*   - - 1. *Who is audited?*     2. *Who conducts the audit?*     3. *What is audited?*     4. *How often?*   *Would it be helpful to have more systems in place to keep track of Sepsis Six performance?*  *Are there any specific meetings where Sepsis Six performance is reviewed? If yes –*   - - 1. *who attends?*     2. *How often?*     3. *Do you attend?*   *Are they useful?* |  |
| 26 | Are you aware of any action plans that are in place at this hospital to improve performance of the Sepsis Six? |  |  |
| 27 | Have you changed anything yourself to improve performance of the Sepsis Six? |  |  |
| **INTENTIONS** | | | |
| 28 | To what extent do you intend to (continue to) perform the Sepsis Six in daily clinical practice? |  |  |
| **OPTIMISM** | | | |
| 29 | How optimistic or pessimistic are you that improving performance of the Sepsis Six holds the potential to improve patient care in the future? |  |  |
| **BELIEFS IN CAPABILITIES** | | | |
|  |  |  | **note: see subprompts for Questions 2 and 4* |
