# Supplementary material for: Barriers and facilitators towards implementing the Sepsis Six care bundle (BLISS-1): a mixed methods investigation using the theoretical domains framework
Source: Scand J Trauma Resusc Emerg Med. 2017 Sep 19;25:96. doi: 10.1186/s13049-017-0437-2 (PMC5606082; doi:10.1186/s13049-017-0437-2)
Supplement: Supplementary file 2 — Barriers and Levers to Implementing the Sepsis Six (BLISS) Questionnaire. (DOCX 67 kb) [file 13049_2017_437_MOESM2_ESM.docx]

**Barriers and Levers to Implementing the Sepsis Six (BLISS)**

Dear All,

We wish to understand how the Sepsis Six care bundle is being implemented. We hope to understand what factors make it easier or more difficult to implement the Sepsis Six, so that we may identify ways to improve the care we deliver to our patients.

We would therefore be very grateful if you could share your views with us, and contribute to this improvement process.   

We would like to invite you to take part in this questionnaire. You are free to stop at any time and your responses will be discarded. The questionnaire will be completely anonymous. By completing and submitting the questionnaire you consent to the storage and use of the collected data for the purposes of the study.

This questionnaire should take approximately 10 minutes to complete.

Each question consists of two opposing opinion statements, and a scale between them. Please select where on the scale you agree with most – or the middle if you agree with neither, or indeed both statements equally. You will then be asked how important you think the issue described in the statement is to your overall performance of the Sepsis Six.

Please answer every question.

Many thanks for your contribution.

Contact for queries:
*Dr Neil Roberts, Anaesthetic and Critical Care Trainee, Royal Cornwall Hospital, Truro
Email: neil.roberts8@nhs.net*

**Please select your role:**

- Consultant or Associate Specialist
- Junior doctor (any non-Consultant or Associate Specialist)
- Registered Nurse

**Please select the main area you work in:**

- Accident & Emergency
- Acute Medical Admissions
- Acute Surgical Admissions

**How many years have you been in this role in your current Trust?**

| Which of these statements do you agree with more? (select appropriate place on the scale) | | | | | | | | How important is this issue to your delivery of the Sepsis Six? | | | | |
| --- | --- | --- | --- | --- | --- | --- | --- | --- | --- | --- | --- | --- |
| **1** | **I am NOT aware of what the Sepsis Six involves** | **1** | **2** | **3** | **4** | **5** | **I AM aware of what the Sepsis Six involves** | **Very unimportant 1** | **Unimportant**  **2** | **No opinion 3** | **Important 4** | **Very important 5** |

**What Trust do you work at?**

The Sepsis Six is a care bundle to be delivered to patients with suspected sepsis.

It consists of:

1. Blood cultures 4. Oxygen

2. Antibiotics 5. Measurement of lactate

3. Intravenous fluids 6. Measurement of urine output

| Which of these statements do you agree with more? (select appropriate place on the scale) | | | | | | | | How important is this issue to your delivery of the Sepsis Six? | | | | |  |  |  |  |  |  |  |  |  |  |  |
| --- | --- | --- | --- | --- | --- | --- | --- | --- | --- | --- | --- | --- | --- | --- | --- | --- | --- | --- | --- | --- | --- | --- | --- |
| **2** | **I do NOT have the necessary skills to perform the Sepsis Six** | **1** | **2** | **3** | **4** | **5** | **I HAVE the necessary skills to perform the Sepsis Six** | **Very unimportant 1** | **Unimportant**  **2** | **No opinion 3** | **Important 4** | **Very important 5** |  |  |  |  |  |  |  |  |  |  |  |
| **3** | I am NOT aware of the evidence supporting the Sepsis Six | **1** | 2 | 3 | 4 | **5** | I AM aware of the evidence supporting the Sepsis Six | 1 | 2 | 3 | 4 | 5 |  |  |  |  |  |  |  |  |  |  |  |
| **4** | **I do NOT intend to improve my knowledge of the Sepsis Six** | **1** | **2** | **3** | **4** | **5** | **I INTEND to improve my knowledge of the Sepsis Six** | **1** | **2** | **3** | **4** | **5** |  |  |  |  |  |  |  |  |  |  |  |
| **5** | There is INSUFFICIENT staffing to perform the Sepsis Six | **1** | 2 | 3 | 4 | **5** | There is SUFFICIENT staffing to perform the Sepsis Six | 1 | 2 | 3 | 4 | 5 |  |  |  |  |  |  |  |  |  |  |  |
| **6** | **There is INSUFFICIENT provision of training required to perform the Sepsis Six** | **1** | **2** | **3** | **4** | **5** | **There is SUFFICIENT provision of training required to perform the Sepsis Six** | **1** | **2** | **3** | **4** | **5** |  |  |  |  |  |  |  |  |  |  |  |
| **7** | It's DIFFICULT to remember all the steps of the Sepsis Six in day-to-day clinical practice | **1** | 2 | 3 | 4 | **5** | It's EASY to remember all the steps of the Sepsis Six in day-to-day clinical practice | 1 | 2 | 3 | 4 | 5 |  |  |  |  |  |  |  |  |  |  |  |
| **8** | **Sepsis Six performance is NOT audited regularly in my department** | **1** | **2** | **3** | **4** | **5** | **Sepsis Six performance IS audited regularly in my department** | **1** | **2** | **3** | **4** | **5** |  |  |  |  |  |  |  |  |  |  |  |
| **9** | I OFTEN miss sepsis | **1** | 2 | 3 | 4 | **5** | I RARELY miss sepsis | 1 | 2 | 3 | 4 | 5 |  |  |  |  |  |  |  |  |  |  |  |
| **10** | **There are INSUFFICIENT tools in use to guide & track Sepsis Six performance in individual patients** | **1** | **2** | **3** | **4** | **5** | **There are SUFFICIENT tools in use to guide & track Sepsis Six performance in individual patients** | **1** | **2** | **3** | **4** | **5** |  |  |  |  |  |  |  |  |  |  |  |
| **11** | Regular use of the Sepsis Six does NOT make it easier to remember the steps involved | **1** | 2 | 3 | 4 | **5** | Regular use of the Sepsis Six DOES make it easier to remember the steps involved | 1 | 2 | 3 | 4 | 5 |  |  |  |  |  |  |  |  |  |  |  |
| **12** | **We get INSUFFICIENT feedback on our Sepsis Six performance** | **1** | **2** | **3** | **4** | **5** | **We get SUFFICIENT feedback on our Sepsis Six performance** | **1** | **2** | **3** | **4** | **5** |  |  |  |  |  |  |  |  |  |  |  |
| **13** | The culture within my department HINDERS performance of the Sepsis Six | **1** | 2 | 3 | 4 | **5** | The culture within my department HELPS performance of the Sepsis Six | 1 | 2 | 3 | 4 | 5 |  |  |  |  |  |  |  |  |  |  |  |
| **14** | **Sepsis Six performance is NOT discussed in meetings in my department** | **1** | **2** | **3** | **4** | **5** | **Sepsis Six performance IS discussed in meetings in my department** | **1** | **2** | **3** | **4** | **5** |  |  |  |  |  |  |  |  |  |  |  |
| **15** | There is INSUFFICIENT leadership for improving Sepsis Six performance | **1** | 2 | 3 | 4 | **5** | There is SUFFICIENT leadership for improving Sepsis Six performance | 1 | 2 | 3 | 4 | 5 |  |  |  |  |  |  |  |  |  |  |  |
| **16** | **Involving clinical staff in Sepsis Six performance improvement will NOT lead to greater improvement** | **1** | **2** | **3** | **4** | **5** | **Involving clinical staff in Sepsis Six performance improvement WILL lead to greater improvements** | **1** | **2** | **3** | **4** | **5** |  |  |  |  |  |  |  |  |  |  |  |
| Which of these statements do you agree with more? (select appropriate place on the scale) | | | | | | | | How important is this issue to your delivery of the Sepsis Six? | | | | |  |  |  |  |  |  | 1 | 2 | 3 | 4 | 5 |
| **17** | It is NOT part of my role to decide when to perform the Sepsis Six | **1** | 2 | 3 | 4 | **5** | It IS part of my role to decide when to perform the Sepsis Six | Very unimportant 1 | Unimportant  2 | No opinion 3 | Important 4 | Very important 5 |  |  |  |  |  |  |  |  |  |  |  |
| **18** | **There are NO plans in place to improve Sepsis Six performance at my hospital** | **1** | **2** | **3** | **4** | **5** | **There ARE plans in place to improve Sepsis Six performance at my hospital** | **1** | **2** | **3** | **4** | **5** |  |  |  |  |  |  |  |  |  |  |  |
| **19** | My colleagues' opinions about the Sepsis Six do NOT affect whether I perform it | **1** | 2 | 3 | 4 | **5** | My colleagues' opinions about the Sepsis Six DO affect whether I perform it | 1 | 2 | 3 | 4 | 5 |  |  |  |  |  |  |  |  |  |  |  |
| **20** | **My colleagues do NOT believe that the Sepsis Six is beneficial to patients** | **1** | **2** | **3** | **4** | **5** | **My colleagues DO believe that the Sepsis Six is beneficial to patients** | **1** | **2** | **3** | **4** | **5** |  |  |  |  |  |  |  |  |  |  |  |
| **21** | Performing the Sepsis Six is NOT part of my role | **1** | 2 | 3 | 4 | **5** | Performing the Sepsis Six IS part of my role | 1 | 2 | 3 | 4 | 5 |  |  |  |  |  |  |  |  |  |  |  |
| **22** | **I do NOT feel bad if I do not deliver the Sepsis Six to a septic patient** | **1** | **2** | **3** | **4** | **5** | **I DO feel bad if I do not deliver the Sepsis Six to a septic patient** | **1** | **2** | **3** | **4** | **5** |  |  |  |  |  |  |  |  |  |  |  |
| **23** | I do NOT feel able to escalate when I am concerned about a patient who may need the Sepsis Six | **1** | 2 | 3 | 4 | **5** | I DO feel able to escalate when I am concerned about a patient who may need the Sepsis Six | 1 | 2 | 3 | 4 | 5 |  |  |  |  |  |  |  |  |  |  |  |
| **24** | **Having a local sepsis 'champion' would NOT improve performance of the Sepsis Six** | **1** | **2** | **3** | **4** | **5** | **Having a local sepsis 'champion' WOULD improve performance of the Sepsis Six** | **1** | **2** | **3** | **4** | **5** |  |  |  |  |  |  |  |  |  |  |  |
| **25** | It is NOT part of my role to identify septic patients | **1** | 2 | 3 | 4 | **5** | It IS part of my role to identify septic patients | 1 | 2 | 3 | 4 | 5 |  |  |  |  |  |  |  |  |  |  |  |
| **26** | **There is INSUFFICIENT time to perform the Sepsis Six** | **1** | **2** | **3** | **4** | **5** | **There is SUFFICIENT time to perform the Sepsis Six** | **1** | **2** | **3** | **4** | **5** |  |  |  |  |  |  |  |  |  |  |  |
| **27** | There is SLOW turnover of medical/nursing staff in areas looking after septic patients | **1** | 2 | 3 | 4 | **5** | There is RAPID turnover of medical/nursing staff in areas looking after septic patients | 1 | 2 | 3 | 4 | 5 |  |  |  |  |  |  |  |  |  |  |  |
| **28** | **Delivering the Sepsis Six quickly does NOT increase how much benefit it has** | **1** | **2** | **3** | **4** | **5** | **Delivering the Sepsis Six quickly DOES increase the benefit it has** | **1** | **2** | **3** | **4** | **5** |  |  |  |  |  |  |  |  |  |  |  |
| **29** | It is NOT part of my role to improve Sepsis Six performance through leadership & support | **1** | 2 | 3 | 4 | **5** | It IS part of my role to improve Sepsis Six performance through leadership & support | 1 | 2 | 3 | 4 | 5 |  |  |  |  |  |  |  |  |  |  |  |
| **30** | **There are some steps in the Sepsis Six which I am NOT ALLOWED to perform** | **1** | **2** | **3** | **4** | **5** | **I am ALLOWED to perform all steps in the Sepsis Six** | **1** | **2** | **3** | **4** | **5** |  |  |  |  |  |  |  |  |  |  |  |
| **31** | There is INSUFFICIENT equipment / medication to perform the Sepsis Six | **1** | 2 | 3 | 4 | **5** | There is SUFFICIENT equipment / medication to perform the Sepsis Six | 1 | 2 | 3 | 4 | 5 |  |  |  |  |  |  |  |  |  |  |  |
| **32** | **There are INSUFFICIENT beds available in my department to look after septic patients** | **1** | **2** | **3** | **4** | **5** | **There are SUFFICIENT beds available in my department to look after septic patients** | **1** | **2** | **3** | **4** | **5** |  |  |  |  |  |  |  |  |  |  |  |
| **33** | When uncertain about diagnosis I WAIT FOR CONFIRMATION of sepsis before performing the Sepsis Six | **1** | 2 | 3 | 4 | **5** | When uncertain about diagnosis I PERFORM the Sepsis Six rather than miss treating potential sepsis | 1 | 2 | 3 | 4 | 5 |  |  |  |  |  |  |  |  |  |  |  |
| Which of these statements do you agree with more? (select appropriate place on the scale) | | | | | | | | How important is this issue to your delivery of the Sepsis Six? | | | | |  |  |  |  |  |  |  |  |  |  |  |
| **34** | **The equipment I need to perform the Sepsis Six does NOT work or works poorly** | **1** | **2** | **3** | **4** | **5** | **The equipment I need to perform the Sepsis Six DOES work well** | **Very unimportant 1** | **Unimportant**  **2** | **No opinion 3** | **Important 4** | **Very important 5** |  |  |  |  |  |  |  |  |  |  |  |
| **35** | I do NOT intend to continue to perform the Sepsis Six on septic patients | **1** | 2 | 3 | 4 | **5** | I DO intend to continue to perform the Sepsis Six on septic patients | 1 | 2 | 3 | 4 | 5 |  |  |  |  |  |  |  |  |  |  |  |
| **36** | **Septic patients are RARELY managed in an appropriate location** | **1** | **2** | **3** | **4** | **5** | **Septic patients are ALWAYS managed in an appropriate location** | **1** | **2** | **3** | **4** | **5** |  |  |  |  |  |  |  |  |  |  |  |
| **37** | Performing the steps in the Sepsis Six does NOT improve patient outcomes | **1** | 2 | 3 | 4 | **5** | Performing the steps in the Sepsis Six DOES improve patient outcomes | 1 | 2 | 3 | 4 | 5 |  |  |  |  |  |  |  |  |  |  |  |
| **38** | **Sepsis Six performance at this hospital will NOT improve** | **1** | **2** | **3** | **4** | **5** | **Sepsis Six performance this this hospital WILL improve** | **1** | **2** | **3** | **4** | **5** |  |  |  |  |  |  |  |  |  |  |  |
| **39** | Overall, the RISKS of performing the Sepsis Six outweigh the benefits | **1** | 2 | 3 | 4 | **5** | Overall, the BENEFITS of performing the Sepsis Six outweigh the risks | 1 | 2 | 3 | 4 | 5 |  |  |  |  |  |  |  |  |  |  |  |
| **40** | **I am UNLIKELY to complete all steps of the Sepsis Six if I think the patient is well** | **1** | **2** | **3** | **4** | **5** | **I am LIKELY to complete all steps of the Sepsis Six even if I think the patient is well** | **1** | **2** | **3** | **4** | **5** |  |  |  |  |  |  |  |  |  |  |  |
| **41** | The RISKS of performing the Sepsis Six outweigh the benefits in CERTAIN patient groups | **1** | 2 | 3 | 4 | **5** | The BENEFITS of performing the Sepsis Six outweigh the risks in ALL patient groups | 1 | 2 | 3 | 4 | 5 |  |  |  |  |  |  |  |  |  |  |  |
| **42** | **There is POOR teamwork when looking after septic patients** | **1** | **2** | **3** | **4** | **5** | **There is GOOD teamwork when looking after septic patients** | **1** | **2** | **3** | **4** | **5** |  |  |  |  |  |  |  |  |  |  |  |
| **43** | Early and regular reassessment of patients requiring the Sepsis Six has NO effect on outcomes | **1** | 2 | 3 | 4 | **5** | Early and regular reassessment of patients requiring the Sepsis Six gives the BEST outcomes | 1 | 2 | 3 | 4 | 5 |  |  |  |  |  |  |  |  |  |  |  |
| **44** | **The hospital is NOT formally rewarded for good Sepsis Six performance** | **1** | **2** | **3** | **4** | **5** | **The hospital IS formally rewarded for good Sepsis Six performance** | **1** | **2** | **3** | **4** | **5** |  |  |  |  |  |  |  |  |  |  |  |
| **45** | I am NOT confident performing the Sepsis Six | **1** | 2 | 3 | 4 | **5** | I AM confident performing the Sepsis Six | 1 | 2 | 3 | 4 | 5 |  |  |  |  |  |  |  |  |  |  |  |
| **46** | **I do NOT prioritise performing the Sepsis Six on a septic patient over other tasks** | **1** | **2** | **3** | **4** | **5** | **I DO prioritise performing the Sepsis Six on a septic patient over other tasks** | **1** | **2** | **3** | **4** | **5** |  |  |  |  |  |  |  |  |  |  |  |
| **47** | Some of the steps in the Sepsis Six are MORE DIFFICULT to perform than others | **1** | 2 | 3 | 4 | **5** | The steps in the Sepsis Six are EQUALLY EASY OR DIFFICULT to perform | 1 | 2 | 3 | 4 | 5 |  |  |  |  |  |  |  |  |  |  |  |
| **48** | **SOME steps in the Sepsis Six are more or less important than others** | **1** | **2** | **3** | **4** | **5** | **ALL steps in the Sepsis Six are equally important** | **1** | **2** | **3** | **4** | **5** |  |  |  |  |  |  |  |  |  |  |  |
| **49** | There is POOR communication between members of the team looking after septic patients | **1** | 2 | 3 | 4 | **5** | There is GOOD communication between members of the team looking after septic patients | 1 | 2 | 3 | 4 | 5 |  |  |  |  |  |  |  |  |  |  |  |
| **50** | **We provide POOR sepsis care at this hospital** | **1** | **2** | **3** | **4** | **5** | **We provide GOOD sepsis care at this hospital** | **1** | **2** | **3** | **4** | **5** |  |  |  |  |  |  |  |  |  |  |  |
|  |  |  |  |  |  |  |  |  |  |  |  |  |  |  |  |  |  |  |  |  |  |  |  |
| Which of these statements do you agree with more? (select appropriate place on the scale) | | | | | | | | **How important is this issue to your delivery of the Sepsis Six?** | | | | |  |  |  |  |  |  |  |  |  |  |  |
| **51** | I do NOT have a time-based goal for completing the Sepsis Six on septic patients | **1** | 2 | 3 | 4 | **5** | My goal is to complete the Sepsis Six within an HOUR on all septic patients | Very unimportant 1 | Unimportant  2 | No opinion 3 | Important 4 | Very important 5 |  |  |  |  |  |  |  |  |  |  |  |
| **52** | **Increasing Sepsis Six performance will NOT improve patient care** | **1** | **2** | **3** | **4** | **5** | **Increasing Sepsis Six performance WILL improve patient care** | **1** | **2** | **3** | **4** | **5** |  |  |  |  |  |  |  |  |  |  |  |
| **53** | Individuals are NOT formally rewarded for good Sepsis Six performance | **1** | 2 | 3 | 4 | **5** | Individuals ARE formally rewarded for good Sepsis Six performance | 1 | 2 | 3 | 4 | 5 |  |  |  |  |  |  |  |  |  |  |  |
| **54** | **I do NOT feel anxious/stressed when treating septic patients** | **1** | **2** | **3** | **4** | **5** | **I DO feel anxious/stressed when treating septic patients** | **1** | **2** | **3** | **4** | **5** |  |  |  |  |  |  |  |  |  |  |  |

**Thank you for taking the time to complete this survey**

**Please place the completed form in one of the collection containers.**

**If you would like any feedback or further information on the survey please provide your email below:**

Contact for queries:

Dr Neil Roberts, Anaesthetic and Critical Care Trainee, Royal Cornwall Hospital, Truro

Email: neil.roberts8@nhs.net
