# Supplementary material for: Barriers and facilitators towards implementing the Sepsis Six care bundle (BLISS-1): a mixed methods investigation using the theoretical domains framework
Source: Scand J Trauma Resusc Emerg Med. 2017 Sep 19;25:96. doi: 10.1186/s13049-017-0437-2 (PMC5606082; doi:10.1186/s13049-017-0437-2)
Supplement: Supplementary file 3 — Table demonstrating belief statement occurrence and data saturation in analyzed interviews (Participants 1–10) with absence of new themes in Participant 11. (DOCX 30 kb) [file 13049_2017_437_MOESM3_ESM.docx]

**Additional file 3. Table demonstrating belief statement occurrence and data saturation in analyzed interviews (Participants 1-10) with absence of new themes in Participant 11.**

| **DOMAIN** | **THEME** | **PARTICIPANT** | | | | | | | | | | |
| --- | --- | --- | --- | --- | --- | --- | --- | --- | --- | --- | --- | --- |
|  |  | **1** | **2** | **3** | **4** | **5** | **6** | **7** | **8** | **9** | **10** | **11** |
| **KNOWLEDGE** | I know/do not know what the Sepsis Six involves | x | x | x | x | x | x | x | x | x | x | x |
|  | My colleagues do/do not know what the Sepsis Six involves | x | x | x | x | x | x | x | x | x | x | x |
|  | I am aware/not aware of the evidence behind the Sepsis Six | x | x | x | x | x | x | x | x | x | x | x |
|  | My colleagues are aware/not aware of the evidence behind the Sepsis Six |  | x |  | x |  |  |  |  |  |  |  |
|  | People would give better Sepsis Six performance if they were more aware of the later complications of poorly managed sepsis |  |  |  |  |  |  |  | x |  |  | x |
|  | Having knowledge and understanding of the Sepsis Six does/does not influence the likelihood of it being performed |  | x | x | x | x | x | x | x | x | x | x |
| **SKILLS** | I do/do not have the skills to perform the Sepsis Six | x | x | x | x | x |  | x | x | x | x | x |
|  | My colleagues do/do not have the skills to perform the Sepsis Six |  | x | x | x | x | x | x | x | x | x | x |
|  | There is/is insufficient provision of training and assessment in the skills required to perform the Sepsis Six | x | x | x | x |  | x | x | x | x | x |  |
| **MEMORY, ATTENTION AND DECISIONS** | It's easy/difficult to remember the 6 steps in clinical practice | x | x | x | x | x | x | x | x | x | x | x |
|  | The decision to start the Sepsis Six is not made because sepsis is not recognised |  | x | x | x | x | x |  | x | x | x | x |
|  | Regular use of the Sepsis Six makes it easier to remember the steps |  | x | x | x | x | x |  |  | x | x | x |
| **BEHAVIORAL REGULATION** | Sepsis Six performance is (not) monitored or audited regularly in my department | x | x | x | x | x | x | x | x | x | x | x |
|  | I/we get insufficient feedback on our Sepsis Six performance | x | x | x | x | x | x | x |  | x | x | x |
|  | There are sufficient tools in place to help guide and track Sepsis Six performance in individual patients | x | x | x | x | x | x | x | x | x | x | x |
|  | Improving sepsis care and Sepsis Six performance is (not) discussed in regular meetings in my department | x |  | x | x | x |  |  |  | x |  | x |
|  | Sepsis Six performance improves if we are involved in the quality improvement process |  |  |  |  | x |  |  | x |  | x |  |
|  | There are (no) action plans to improve Sepsis Six performance | x | x | x | x |  |  |  |  |  |  | x |
| **SOCIAL INFLUENCES** | My colleagues opinions do/do not affect my performance of the Sepsis Six | x | x | x | x | x | x | x | x | x | x | x |
|  | My Colleagues do/do not believe that the Sepsis Six is beneficial to patient care | x | x | x | x | x | x | x |  | x | x |  |
|  | Departmental culture facilitates/hinders performance of the Sepsis Six |  |  | x |  | x |  |  | x |  | x | x |
|  | There is insufficient leadership to improve Sepsis Six performance |  |  | x | x | x | x |  | x | x | x | x |
|  | Healthcare workers do/do not feel able to escalate up the hierarchy |  |  | x |  | x |  |  |  | x | x |  |
|  | Having a Sepsis “Champion” would/would not improve performance of the Sepsis Six |  |  | x |  |  | x |  | x |  |  |  |
| **SOCIAL AND PROFESSIONAL ROLE** | Performing the steps in the Sepsis Six is (not) my role | x | x | x | x | x | x | x | x | x | x | x |
|  | Performing all steps in the Sepsis Six is (not) my colleagues' role |  |  |  |  |  | x |  |  | x | x | x |
|  | It is my/my colleagues' role (doctor/nurse/HCA) to identify septic patients |  | x |  | x | x | x |  |  |  | x | x |
|  | It is my role to decide when to perform the Sepsis Six |  | x | x |  |  |  | x |  |  | x | x |
|  | There is high turnover of medical/nursing staff in areas looking after septic patients |  |  | x | x |  |  |  |  |  | x |  |
|  | My role is to improve Sepsis Six performance through non-clinical factors (leadership, support, supervision) |  |  | x | x |  | x |  |  | x |  | x |
|  | There are some steps in the Sepsis Six which I/my colleagues do not/are not allowed to perform |  | x |  | x |  | x | x | x | x |  |  |
|  | Non-clinical staff (eg bed management) put pressure on clinical staff to prioritise tasks other than Sepsis Six |  |  |  |  | x |  |  |  |  |  | x |
|  | Staff should be empowered to improve their role in Sepsis Six performance |  | x |  | x |  |  |  |  |  |  |  |
| **ENVIRONMENT, CONTEXT AND RESOURCES** | I do (not) have sufficient resources (staff; time; equipment; medicines; bed) to perform the Sepsis Six in one hour. | x | x | x | x | x | x | x | x | x | x | x |
|  | The equipment I have does/doesn’t work |  |  | x | x | x | x | x | x | x | x | x |
|  | The layout of the hospital hinders/helps my performance of the Sepsis six in one hour (patient location, equipment, medicine). |  | x |  | x | x | x | x | x | x | x | x |
| **BELIEFS IN CONSEQUENCES** | Performing the steps in the Sepsis Six improves patient outcomes | x | x | x | x | x | x | x | x | x | x | x |
|  | The benefits of performing the Sepsis Six outweigh the risks |  | x | x | x |  | x | x | x | x | x | x |
|  | The benefits vs risks of performing the Sepsis Six (or some parts of it) are (not) different in certain patient groups | x | x | x | x | x | x | x | x | x | x | x |
|  | The quicker the steps can be delivered, the more impact they have | x | x |  |  |  | x |  | x | x |  | x |
|  | Early and regular reassessment of patients requiring the Sepsis Six gives the best outcomes |  |  |  |  | x |  |  |  |  |  | x |
| **BELIES IN CAPABILITIES** | I am (not) confident performing the steps in the Sepsis Six | x | x | x | x |  | x | x | x | x | x | x |
|  | My colleagues are (not) confident performing the steps in the Sepsis Six |  | x | x |  | x | x | x |  | x | x | x |
|  | Some of the Sepsis Six steps are more difficult than others to achieve (urine output, cultures, antibiotics) | x | x | x | x | x | x | x |  | x | x | x |
|  | There is good/poor communication and teamwork between members of the team looking after septic patients | x | x | x | x | x | x | x | x | x | x | x |
|  | We provide good sepsis care at this hospital |  |  |  |  | x |  |  |  |  |  |  |
|  | I am confident looking after sick septic patients |  | x |  |  |  |  |  |  |  |  | x |
| **INTENTIONS** | I (don't) prioritise performing the Sepsis Six on a septic patient over other tasks | x | x | x | x | x | x | x | x | x | x | x |
|  | I intend to improve my knowledge of the Sepsis Six | x | x |  |  |  |  |  |  |  |  |  |
|  | I intend to continue to perform the Sepsis Six on septic patients |  | x |  | x | x | x | x | x | x | x | x |
|  | I am more likely to complete all steps of the Sepsis Six if I think the patient is sick/less likely if they are well |  | x | x |  |  | x | x |  | x |  |  |
|  | Sometimes I choose (not) to complete the full Sepsis Six because the risks and benefits are different for that patient/situation. |  |  | x | x | x | x | x |  | x | x | x |
|  | My colleagues (don't) prioritise performing the Sepsis Six on a septic patients over other tasks |  |  | x |  |  |  |  |  | x |  | x |
|  | Some steps in the Sepsis Six are more/less important than others |  | x | x | x |  | x | x |  |  | x | x |
|  | I (don't) perform the Sepsis Six despite not having a confirmed diagnosis because I (don't) believe the risks of undertreating sepsis outweigh the risks of performing the Sepsis Six |  |  |  |  | x |  |  |  | x | x |  |
| **GOALS** | I work towards a goal that the Sepsis Six should be completed and documented within an hour on all septic patients. | x | x | x | x | x | x | x | x | x | x | x |
|  | The hospital has/does not have a goal of improving Sepsis Six compliance | x | x | x | x | x | x | x | x | x | x |  |
| **OPTIMISM** | Sepsis Six compliance at this hospital will (not) improve |  |  |  | x | x | x |  |  |  | x | x |
|  | Increasing Sepsis Six compliance will improve patient care | x | x | x | x | x |  | x | x | x | x | x |
| **REINFORCEMENT** | Individuals are not formally rewarded or punished for (failing to) complete the Sepsis Six | x | x | x | x | x | x | x | x | x | x | x |
|  | The department or hospital is (not) formally rewarded or punished for (failing to) complete the Sepsis Six | x | x | x | x | x |  |  | x |  |  | x |
| **EMOTIONS** | I get emotionally affected negatively/positively by managing septic patients |  | x | x | x | x | x | x | x | x | x | x |
|  | If we are affected emotionally (eg stressed, excited, fatigued) it leads to better/worse clinical performance when looking after septic patients | x | x | x | x | x | x | x | x |  | x | x |
|  | I feel good if I deliver the Sepsis Six/bad if I don't deliver the Sepsis Six to a septic patient | x |  |  | x | x |  |  | x |  |  |  |
