# Supplementary material for: Barriers and facilitators towards implementing the Sepsis Six care bundle (BLISS-1): a mixed methods investigation using the theoretical domains framework
Source: Scand J Trauma Resusc Emerg Med. 2017 Sep 19;25:96. doi: 10.1186/s13049-017-0437-2 (PMC5606082; doi:10.1186/s13049-017-0437-2)
Supplement: Supplementary file 4 — Complete results table with median scores and interquartile ranges (IQR). (DOCX 225 kb) [file 13049_2017_437_MOESM4_ESM.docx]

**Supplementary Digital Content 4. Complete results table with median scores and interquartile ranges (IQR).**

| **Strong agreement with barrier statement/**  **Very unimportant**  **(0)** | **Strong agreement with facilitator statement/**  **Very important**  **(5)** | **Overall median** | *Overall IQR* | **ED median** | *ED IQR* | **MAU median** | *MAU IQR* | **SAU median** | *SAU IQR* | **Cons median** | *Cons IQR* | **Jun median** | *Jun IQR* | **Nurse median** | *Nurse IQR* | **ED Cons median** | *ED Cons IQR* | **ED Jun median** | *ED Jun IQR* | **ED Nurse median** | *ED Nurse IQR* | **MAU Cons median** | *MAU Cons IQR* | **MAU Jun median** | *MAU Jun IQR* | **MAU Nurse median** | *MAU Nurse IQR* | **SAU Cons median** | *SAU Cons IQR* | **SAU Jun median** | *SAU Jun IQR* | **SAU Nurse median** | *SAU Nurse IQR* |
| --- | --- | --- | --- | --- | --- | --- | --- | --- | --- | --- | --- | --- | --- | --- | --- | --- | --- | --- | --- | --- | --- | --- | --- | --- | --- | --- | --- | --- | --- | --- | --- | --- | --- |
| Baseline agreement score (group median agreement score for overall questionnaire) | | **5** | | **5** | | **5** | | **4** | | **4** | | **4** | | **5** | | **5** | | **4** | | **5** | | **4** | | **5** | | **5** | | **4** | | **4** | | **4** | |
| Baseline importance score (group median importance score for overall questionnaire) | | **5** | | **5** | | **5** | | **4** | | **4** | | **4** | | **5** | | **5** | | **4** | | **5** | | **4** | | **4** | | **5** | | **4** | | **4** | | **5** | |
| I am NOT aware of what the Sepsis Six involves | I AM aware of what the Sepsis Six involves | **5** | *0* | **5** | *0* | **5** | *0* | **5** | *0* | **5** | *0* | **5** | *0* | **5** | *0* | **5** | *0* | **5** | *0* | **5** | *0* | **5** | *0* | **5** | *0* | **5** | *0* | **5** | *0* | **5** | *0* | **5** | *0.25* |
| Very unimportant | Very important | **5** | *1* | **5** | *0* | **5** | *1* | **5** | *1* | **4** | *1* | **5** | *1* | **5** | *0* | **5** | *0* | **4** | *0.5* | **5** | *0* | **4** | *1.5* | **5** | *0* | **5** | *0* | **4** | *0.5* | **5** | *0.25* | **5** | *0* |
| I do NOT have the necessary skills to perform the Sepsis Six | I HAVE the necessary skills to perform the Sepsis Six | **5** | *0* | **5** | *0* | **5** | *0* | **5** | *1* | **5** | *0* | **5** | *0* | **5** | *1* | **5** | *0* | **5** | *0* | **5** | *0.25* | **5** | *0* | **5** | *0* | **5** | *1* | **5** | *0* | **5** | *0* | **4** | *1* |
| Very unimportant | Very important | **5** | *1* | **5** | *0.75* | **5** | *1* | **5** | *1* | **5** | *1* | **5** | *1* | **5** | *0* | **5** | *1* | **4** | *1* | **5** | *0* | **4** | *2* | **5** | *0.5* | **5** | *0* | **5** | *0.5* | **5** | *1* | **4.5** | *1* |
| I am NOT aware of the evidence supporting the Sepsis Six | I AM aware of the evidence supporting the Sepsis Six | **5** | *1* | **5** | *1* | **5** | *1* | **4** | *1* | **4** | *1* | **5** | *1* | **5** | *1* | **4** | *0.5* | **4** | *1.5* | **5** | *1* | **4** | *1* | **5** | *1* | **5** | *0* | **4** | *1* | **4.5** | *1* | **4** | *1.25* |
| Very unimportant | Very important | **4** | *1* | **5** | *1* | **4** | *1* | **4** | *1* | **4** | *1* | **4** | *1* | **5** | *1* | **4** | *2* | **4** | *1* | **5** | *1* | **4** | *2* | **4** | *0.5* | **5** | *0* | **4** | *0.5* | **4** | *1* | **5** | *1* |
| I do NOT intend to improve my knowledge of the Sepsis Six | I INTEND to improve my knowledge of the Sepsis Six | **4** | *2* | **4** | *1.75* | **4** | *2* | **4** | *2* | **3** | *1* | **4** | *2* | **5** | *1* | **4** | *2* | **4** | *1.5* | **5** | *1* | **4** | *1* | **4** | *2* | **5** | *0.75* | **3** | *0.5* | **4** | *2* | **5** | *0* |
| Very unimportant | Very important | **4** | *2* | **4** | *2* | **4** | *2* | **4** | *2* | **3** | *1* | **3.5** | *1* | **5** | *1* | **3** | *1.5* | **3** | *0.5* | **5** | *1* | **4** | *2* | **4** | *1.5* | **5** | *0* | **3** | *1* | **4** | *1.25* | **5** | *0* |
| There is INSUFFICIENT staffing to perform the Sepsis Six | There is SUFFICIENT staffing to perform the Sepsis Six | **3** | *2* | **3** | *1* | **3** | *2* | **4** | *3* | **3** | *3* | **3** | *2* | **3** | *2* | **3** | *1.5* | **3** | *1* | **3** | *1.25* | **3** | *2.5* | **3** | *2* | **3** | *2* | **5** | *2* | **2.5** | *1.25* | **4** | *2* |
| Very unimportant | Very important | **5** | *1* | **5** | *0* | **5** | *1* | **4** | *1* | **5** | *1* | **5** | *1* | **5** | *0* | **5** | *0* | **5** | *0.5* | **5** | *0* | **5** | *1* | **5** | *1* | **5** | *0* | **4** | *0.5* | **5** | *1* | **5** | *1* |
| There is INSUFFICIENT provision of training required to perform the Sepsis Six | There is SUFFICIENT provision of training required to perform the Sepsis Six | **4** | *2* | **4** | *1.75* | **4** | *2* | **4** | *2* | **4** | *2* | **4** | *1* | **4** | *2* | **4** | *0* | **4** | *2* | **4** | *1.25* | **4** | *2* | **4** | *1.5* | **4.5** | *1* | **3** | *2.5* | **5** | *1* | **4** | *1.25* |
| Very unimportant | Very important | **5** | *1* | **5** | *1* | **5** | *1* | **5** | *1* | **4** | *2* | **4** | *1* | **5** | *0* | **5** | *1* | **4** | *1.5* | **5** | *0* | **4** | *2* | **5** | *1* | **5** | *0* | **4** | *1* | **4.5** | *1* | **5** | *1* |
| It's DIFFICULT to remember all the steps of the Sepsis Six in day-to-day clinical practice | It's EASY to remember all the steps of the Sepsis Six in day-to-day clinical practice | **5** | *1* | **5** | *1* | **5** | *1* | **5** | *1* | **5** | *1* | **5** | *0* | **5** | *1* | **5** | *0* | **5** | *0* | **5** | *1* | **5** | *1* | **5** | *0* | **5** | *1* | **5** | *0.5* | **5** | *0* | **4** | *1* |
| Very unimportant | Very important | **5** | *1* | **5** | *1* | **5** | *1* | **5** | *1* | **4** | *2* | **5** | *1* | **5** | *0.75* | **5** | *1* | **4** | *1.5* | **5** | *1* | **4** | *2* | **5** | *1* | **5** | *0* | **4** | *0.5* | **5** | *1* | **5** | *1* |
| Sepsis Six performance is NOT audited regularly in my department | Sepsis Six performance IS audited regularly in my department | **4** | *2* | **5** | *0.75* | **4** | *2* | **3** | *1* | **4** | *2* | **4** | *2* | **5** | *1.75* | **5** | *1* | **4** | *0.5* | **5** | *0* | **4** | *2.5* | **4** | *1* | **4** | *2* | **4** | *2* | **3** | *1.25* | **3** | *1* |
| Very unimportant | Very important | **4** | *2* | **4.5** | *1.75* | **4** | *1.25* | **4** | *1.5* | **4** | *2* | **4** | *1.75* | **4** | *1* | **4** | *1.5* | **3** | *1* | **5** | *1* | **4** | *2* | **4** | *1* | **4** | *1* | **4** | *1.5* | **3** | *1* | **4** | *1.25* |
| I OFTEN miss sepsis | I RARELY miss sepsis | **4** | *1* | **4** | *1* | **4** | *1* | **4** | *1* | **4** | *1* | **4.5** | *1* | **4** | *1* | **4** | *0.5* | **4** | *1.5* | **4** | *1* | **4** | *0.5* | **5** | *1* | **4** | *1* | **5** | *1* | **4.5** | *1* | **4** | *0* |
| Very unimportant | Very important | **5** | *1* | **5** | *1* | **5** | *1* | **5** | *1* | **4** | *1* | **5** | *1* | **5** | *0.75* | **5** | *1* | **4** | *1.5* | **5** | *0* | **4** | *2* | **5** | *1* | **5** | *0.75* | **4** | *0.5* | **5** | *0.25* | **5** | *1* |
| There are INSUFFICIENT tools in use to guide & track Sepsis Six performance in individual patients | There are SUFFICIENT tools in use to guide & track Sepsis Six performance in individual patients | **4** | *2* | **4** | *1.75* | **4** | *2* | **4** | *2* | **4** | *2* | **4** | *2* | **4** | *1.75* | **4** | *2.5* | **4** | *2* | **4.5** | *1* | **3** | *2* | **4** | *2* | **4** | *1.75* | **4** | *1.5* | **4.5** | *1* | **4** | *1* |
| Very unimportant | Very important | **4** | *1* | **5** | *1* | **4** | *2* | **4** | *1* | **4** | *1* | **4** | *2* | **5** | *1* | **4** | *1* | **3** | *1* | **5** | *1* | **3** | *1.5* | **4** | *0* | **5** | *1* | **4** | *0.5* | **4.5** | *1.25* | **4.5** | *1* |
| Regular use of the Sepsis Six does NOT make it easier to remember the steps involved | Regular use of the Sepsis Six DOES make it easier to remember the steps involved | **5** | *0* | **5** | *0* | **5** | *0* | **5** | *1* | **5** | *0* | **5** | *0* | **5** | *0* | **5** | *0* | **5** | *0* | **5** | *0* | **5** | *0* | **5** | *0* | **5** | *0* | **4** | *1* | **5** | *1* | **5** | *0.25* |
| Very unimportant | Very important | **5** | *1* | **5** | *1* | **4.5** | *1* | **4** | *1* | **4** | *1* | **4** | *1* | **5** | *1* | **4** | *0.5* | **4** | *0.5* | **5** | *0.25* | **4** | *1.5* | **5** | *1.5* | **5** | *1* | **3** | *1* | **5** | *1* | **5** | *1* |
| We get INSUFFICIENT feedback on our Sepsis Six performance | We get SUFFICIENT feedback on our Sepsis Six performance | **3** | *3* | **4** | *2* | **3** | *2* | **2** | *2* | **3** | *2* | **3** | *2* | **4** | *3* | **3** | *2* | **3** | *2* | **5** | *1* | **3** | *2* | **3** | *1.5* | **2.5** | *2* | **2** | *2.5* | **2.5** | *2.25* | **2** | *1.25* |
| Very unimportant | Very important | **4** | *2* | **4.5** | *1* | **4** | *2* | **4** | *1* | **4** | *1* | **4** | *1* | **5** | *1* | **4** | *1* | **4** | *1* | **5** | *1* | **4** | *1.5* | **4** | *1.5* | **4.5** | *1* | **4** | *0* | **3** | *1* | **4** | *1.25* |
| The culture within my department HINDERS performance of the Sepsis Six | The culture within my department HELPS performance of the Sepsis Six | **4** | *1* | **4** | *1* | **5** | *1* | **4** | *2* | **4** | *2* | **4** | *1* | **4** | *1* | **4** | *1* | **4** | *1* | **4** | *0.25* | **4** | *2* | **5** | *1* | **5** | *0.75* | **4** | *0.5* | **3.5** | *2* | **4.5** | *1* |
| Very unimportant | Very important | **5** | *1* | **5** | *1* | **5** | *1* | **4** | *1* | **4** | *1* | **4** | *1* | **5** | *1* | **5** | *0.5* | **4** | *1* | **5** | *1* | **4** | *1.5* | **5** | *1* | **5** | *1* | **4** | *0.5* | **4** | *2* | **5** | *1* |
| Sepsis Six performance is NOT discussed in meetings in my department | Sepsis Six performance IS discussed in meetings in my department | **4** | *2* | **5** | *1* | **4** | *2* | **3** | *2* | **4** | *2* | **4** | *2* | **5** | *1* | **5** | *0* | **4** | *1* | **5** | *1* | **4** | *2* | **4** | *2* | **5** | *1* | **3** | *2* | **3** | *2.5* | **4** | *1.25* |
| Very unimportant | Very important | **4** | *1* | **4** | *1* | **4** | *1* | **4** | *1* | **4** | *1* | **4** | *1* | **5** | *1* | **4** | *1* | **3** | *1* | **5** | *1* | **4** | *1.5* | **4** | *0.5* | **5** | *1* | **4** | *0.5* | **4** | *1.25* | **4** | *1* |
| There is INSUFFICIENT leadership for improving Sepsis Six performance | There is SUFFICIENT leadership for improving Sepsis Six performance | **4** | *2* | **4** | *1* | **4** | *1.25* | **4** | *1* | **4** | *2* | **4** | *1* | **4** | *1.75* | **4** | *1* | **4** | *0.5* | **4** | *1.25* | **4** | *2* | **5** | *1* | **5** | *1* | **4** | *1* | **3.5** | *1* | **4** | *1* |
| Very unimportant | Very important | **4** | *1* | **5** | *1* | **5** | *1* | **4** | *1.5* | **4** | *1* | **4** | *1.75* | **5** | *1* | **5** | *1* | **4** | *0.5* | **5** | *1* | **5** | *1.5* | **4** | *1* | **5** | *0.75* | **4** | *0* | **4** | *2* | **4** | *1* |
| Involving clinical staff in Sepsis Six performance improvement will NOT lead to greater improvement | Involving clinical staff in Sepsis Six performance improvement WILL lead to greater improvements | **5** | *1* | **5** | *0* | **5** | *0* | **5** | *1* | **5** | *1* | **5** | *1* | **5** | *0* | **5** | *0.5* | **5** | *0.5* | **5** | *0* | **5** | *0.5* | **5** | *0* | **5** | *0* | **4** | *0.5* | **4.5** | *1* | **5** | *0* |
| Very unimportant | Very important | **5** | *1* | **5** | *1* | **4** | *1* | **4** | *1* | **4** | *1* | **4** | *1.75* | **5** | *1* | **5** | *1* | **4** | *1.5* | **5** | *1* | **4** | *1* | **4** | *1* | **5** | *1* | **4** | *0.5* | **4** | *1.25* | **5** | *0* |
| It is NOT part of my role to decide when to perform the Sepsis Six | It IS part of my role to decide when to perform the Sepsis Six | **5** | *0* | **5** | *0* | **5** | *0* | **5** | *0* | **5** | *0* | **5** | *0* | **5** | *0* | **5** | *0* | **5** | *0* | **5** | *0* | **5** | *1* | **5** | *0* | **5** | *0* | **5** | *0* | **5** | *0* | **5** | *1* |
| Very unimportant | Very important | **5** | *1* | **5** | *1* | **5** | *1* | **5** | *1* | **4** | *1* | **5** | *1* | **5** | *0* | **5** | *1* | **5** | *1* | **5** | *0.25* | **4** | *1* | **5** | *1* | **5** | *0.75* | **4** | *1* | **5** | *1* | **5** | *0* |
| There are NO plans in place to improve Sepsis Six performance at my hospital | There ARE plans in place to improve Sepsis Six performance at my hospital | **4** | *1* | **5** | *1* | **4** | *1* | **4** | *2* | **4** | *2* | **4** | *1.75* | **5** | *1* | **5** | *1* | **4** | *1* | **5** | *1* | **4** | *2* | **4** | *0.5* | **5** | *1* | **4** | *1.5* | **4** | *2* | **5** | *1* |
| Very unimportant | Very important | **5** | *1* | **5** | *1* | **4** | *1* | **4** | *2* | **4** | *2* | **4** | *2* | **5** | *1* | **4** | *1* | **3** | *2* | **5** | *0.25* | **4** | *1.5* | **4** | *1.5* | **5** | *0* | **4** | *1.5* | **4** | *2* | **5** | *1* |
| My colleagues' opinions about the Sepsis Six do NOT affect whether I perform it | My colleagues' opinions about the Sepsis Six DO affect whether I perform it | **2** | *3* | **2.5** | *3* | **3** | *3.25* | **2** | *2.5* | **2** | *3* | **3** | *2* | **2** | *3* | **4** | *2.5* | **4** | *2* | **2** | *3* | **2** | *2.5* | **3** | *2.5* | **1.5** | *3.5* | **1** | *3* | **2** | *1.5* | **3** | *2.25* |
| Very unimportant | Very important | **4** | *2* | **4** | *1.75* | **3.5** | *2* | **4** | *1* | **3** | *1* | **4** | *1* | **5** | *2* | **3** | *2* | **4** | *0.5* | **5** | *1* | **3** | *1* | **4** | *1* | **3.5** | *3* | **3** | *1* | **3** | *1* | **4** | *2* |
| My colleagues do NOT believe that the Sepsis Six is beneficial to patients | My colleagues DO believe that the Sepsis Six is beneficial to patients | **5** | *1* | **5** | *1* | **5** | *1* | **5** | *1* | **5** | *1* | **5** | *1* | **5** | *0* | **5** | *1* | **4** | *1.5* | **5** | *1* | **5** | *1* | **5** | *0.5* | **5** | *0* | **5** | *1.5* | **4.5** | *1* | **5** | *0* |
| Very unimportant | Very important | **4** | *1* | **5** | *1* | **4** | *2* | **4** | *1* | **4** | *1* | **4** | *2* | **5** | *1* | **4** | *1* | **4** | *1* | **5** | *1* | **4** | *2* | **4** | *2* | **5** | *1* | **4** | *0* | **4** | *1.25* | **5** | *0.25* |
| Performing the Sepsis Six is NOT part of my role | Performing the Sepsis Six IS part of my role | **5** | *0* | **5** | *0* | **5** | *0* | **5** | *0.5* | **5** | *1* | **5** | *0* | **5** | *0* | **5** | *0* | **5** | *0* | **5** | *0* | **5** | *1.5* | **5** | *0* | **5** | *0* | **5** | *1* | **5** | *0* | **5** | *1* |
| Very unimportant | Very important | **5** | *1* | **5** | *1* | **5** | *2* | **5** | *1* | **4** | *1* | **5** | *1* | **5** | *0* | **5** | *0.5* | **4** | *0.5* | **5** | *0* | **4** | *2* | **5** | *1* | **5** | *0.75* | **4** | *0* | **5** | *1* | **5** | *0* |
| I do NOT feel bad if I do not deliver the Sepsis Six to a septic patient | I DO feel bad if I do not deliver the Sepsis Six to a septic patient | **5** | *0* | **5** | *0* | **5** | *0* | **5** | *1* | **5** | *1* | **5** | *0* | **5** | *0* | **5** | *0* | **5** | *0.5* | **5** | *0.25* | **5** | *1.5* | **5** | *0* | **5** | *0* | **4** | *1* | **5** | *0* | **5** | *0.25* |
| Very unimportant | Very important | **5** | *1* | **5** | *1* | **5** | *1* | **4** | *1* | **4** | *1* | **5** | *1* | **5** | *0* | **4** | *1* | **4** | *1* | **5** | *0.25* | **4** | *2* | **5** | *1* | **5** | *0* | **4** | *0* | **4** | *1* | **5** | *0.25* |
| I do NOT feel able to escalate when I am concerned about a patient who may need the Sepsis Six | I DO feel able to escalate when I am concerned about a patient who may need the Sepsis Six | **5** | *0* | **5** | *0* | **5** | *0* | **5** | *0* | **5** | *0* | **5** | *0* | **5** | *0* | **5** | *0* | **5** | *0.5* | **5** | *0* | **5** | *0* | **5** | *0* | **5** | *0* | **5** | *0* | **5** | *1* | **5** | *0* |
| Very unimportant | Very important | **5** | *1* | **5** | *1* | **5** | *1* | **5** | *1* | **4** | *1* | **4.5** | *1* | **5** | *0* | **5** | *1* | **4** | *1* | **5** | *0* | **4** | *2* | **5** | *1* | **5** | *0* | **4** | *0* | **4.5** | *1* | **5** | *0* |
| Having a local sepsis 'champion' would NOT improve performance of the Sepsis Six | Having a local sepsis 'champion' WOULD improve performance of the Sepsis Six | **4** | *3* | **4** | *2* | **3.5** | *3* | **3** | *2.5* | **4** | *4* | **3** | *1.75* | **4** | *2* | **5** | *0.5* | **2** | *1.5* | **4** | *2* | **4** | *3* | **3** | *2* | **4.5** | *2* | **1** | *3* | **2** | *1.25* | **4** | *2* |
| Very unimportant | Very important | **4** | *2* | **4** | *2* | **3.5** | *2* | **3** | *1.5* | **4** | *1* | **3** | *1* | **4** | *2* | **4** | *1* | **3** | *1.5* | **4** | *2* | **4** | *1.5* | **3** | *1* | **4** | *2* | **3** | *3* | **3** | *1* | **4.5** | *1* |
| It is NOT part of my role to identify septic patients | It IS part of my role to identify septic patients | **5** | *0* | **5** | *0* | **5** | *0* | **5** | *0* | **5** | *0* | **5** | *0* | **5** | *0* | **5** | *0* | **5** | *0* | **5** | *0* | **5** | *0* | **5** | *0* | **5** | *0* | **5** | *0* | **5** | *0* | **5** | *0* |
| Very unimportant | Very important | **5** | *1* | **5** | *0* | **5** | *1* | **5** | *1* | **5** | *1* | **5** | *1* | **5** | *0* | **5** | *0.5* | **4** | *1* | **5** | *0* | **4** | *1.5* | **5** | *1* | **5** | *0* | **4** | *1* | **5** | *0.25* | **5** | *0* |
| There is INSUFFICIENT time to perform the Sepsis Six | There is SUFFICIENT time to perform the Sepsis Six | **4** | *1* | **3** | *2* | **4** | *1.25* | **4** | *1* | **3** | *2* | **4** | *1* | **4** | *1* | **3** | *1.5* | **3** | *2* | **3** | *2* | **3** | *2.5* | **4** | *1* | **4** | *1* | **5** | *0.5* | **4** | *1.5* | **4** | *0.25* |
| Very unimportant | Very important | **5** | *1* | **5** | *0* | **5** | *1* | **5** | *1* | **5** | *1* | **5** | *1* | **5** | *0* | **5** | *0* | **5** | *0.5* | **5** | *0* | **4** | *2* | **5** | *1* | **5** | *0.75* | **4** | *1* | **5** | *1* | **5** | *0* |
| There is RAPID turnover of medical/nursing staff in areas looking after septic patients | There is SLOW turnover of medical/nursing staff in areas looking after septic patients | **2** | *1* | **2** | *2* | **1** | *1* | **2** | *1.5* | **2** | *1* | **1** | *1* | **2** | *2* | **2** | *0* | **1** | *1* | **2** | *2* | **2** | *1.5* | **1** | *1* | **1.5** | *1* | **2** | *1* | **1** | *0* | **2** | *1.25* |
| Very unimportant | Very important | **4** | *1* | **4** | *1.75* | **4** | *1* | **4** | *1* | **4** | *1* | **4** | *1* | **4** | *1* | **5** | *1* | **4** | *1* | **5** | *2* | **4** | *1.5* | **4** | *1* | **4** | *1* | **4** | *0.5* | **5** | *1* | **4** | *1* |
| Delivering the Sepsis Six quickly does NOT increase how much benefit it has | Delivering the Sepsis Six quickly DOES increase the benefit it has | **5** | *0* | **5** | *0* | **5** | *0* | **5** | *0* | **5** | *0* | **5** | *0* | **5** | *0* | **5** | *0* | **5** | *0.5* | **5** | *0* | **5** | *0* | **5** | *0* | **5** | *0* | **5** | *0* | **5** | *0* | **5** | *0* |
| Very unimportant | Very important | **5** | *1* | **5** | *0* | **5** | *1* | **5** | *1* | **5** | *1* | **5** | *0.75* | **5** | *0* | **5** | *0.5* | **5** | *0.5* | **5** | *0* | **5** | *1* | **5** | *0* | **5** | *0* | **4** | *1* | **5** | *1* | **5** | *0* |
| It is NOT part of my role to improve Sepsis Six performance through leadership & support | It IS part of my role to improve Sepsis Six performance through leadership & support | **5** | *1* | **5** | *1* | **5** | *1* | **5** | *1* | **5** | *1* | **4** | *1* | **5** | *0* | **5** | *0* | **4** | *0.5* | **5** | *1* | **5** | *1* | **5** | *2* | **5** | *0* | **5** | *0* | **4** | *1* | **5** | *0* |
| Very unimportant | Very important | **4** | *1* | **5** | *1* | **4** | *1* | **5** | *1* | **4** | *1* | **4** | *1.75* | **5** | *1* | **5** | *1* | **4** | *1* | **5** | *1* | **4** | *1* | **4** | *1.5* | **5** | *1* | **4** | *1* | **4** | *0.25* | **5** | *0* |
| There are some steps in the Sepsis Six which I am NOT ALLOWED to perform | I am ALLOWED to perform all steps in the Sepsis Six | **5** | *2* | **5** | *1.75* | **5** | *2* | **5** | *2* | **5** | *0* | **5** | *0* | **3** | *4* | **5** | *0* | **5** | *1* | **5** | *2* | **5** | *0.5* | **5** | *0* | **2.5** | *3* | **5** | *0* | **5** | *0* | **2.5** | *2.25* |
| Very unimportant | Very important | **5** | *1* | **5** | *1* | **4** | *2* | **5** | *1* | **4** | *2* | **5** | *1* | **5** | *1* | **4** | *1* | **4** | *1* | **5** | *1* | **4** | *2* | **5** | *1* | **4** | *2* | **4** | *0.5* | **5** | *1* | **5** | *1* |
| There is INSUFFICIENT equipment / medication to perform the Sepsis Six | There is SUFFICIENT equipment / medication to perform the Sepsis Six | **4** | *1* | **5** | *1* | **4** | *1.25* | **5** | *1* | **5** | *1* | **5** | *1* | **4** | *1* | **5** | *0.5* | **5** | *0.5* | **4** | *1* | **4** | *1.5* | **5** | *1* | **4** | *1.75* | **5** | *0.5* | **4** | *1* | **4.5** | *1.25* |
| Very unimportant | Very important | **5** | *1* | **5** | *1* | **5** | *1* | **5** | *1* | **4** | *1* | **4.5** | *1* | **5** | *0* | **5** | *1* | **4** | *1* | **5** | *0* | **4** | *1.5* | **5** | *1* | **5** | *0.75* | **4** | *0.5* | **4** | *1* | **5** | *0* |
| There are INSUFFICIENT beds available in my department to look after septic patients | There are SUFFICIENT beds available in my department to look after septic patients | **2** | *2* | **1.5** | *2* | **3** | *3* | **3** | *2* | **1** | *1* | **3** | *2* | **3** | *3* | **1** | *1* | **2** | *1.5* | **1** | *2* | **1** | *1.5* | **3** | *2.5* | **3** | *2* | **1** | *0.5* | **3** | *2* | **4** | *1.25* |
| Very unimportant | Very important | **5** | *1* | **5** | *0* | **5** | *1* | **5** | *1* | **5** | *0* | **5** | *1* | **5** | *0* | **5** | *0* | **4** | *1.5* | **5** | *0* | **5** | *0.5* | **5** | *0.5* | **5** | *1* | **5** | *0.5* | **4** | *1.25* | **5** | *0.25* |
| When uncertain about diagnosis I WAIT FOR CONFIRMATION of sepsis before performing the Sepsis Six | When uncertain about diagnosis I PERFORM the Sepsis Six rather than miss treating potential sepsis | **5** | *1* | **5** | *1* | **4.5** | *1* | **5** | *1* | **5** | *1* | **5** | *1* | **4** | *1* | **5** | *0.5* | **4** | *1* | **4.5** | *2* | **4** | *1* | **5** | *1* | **5** | *1* | **5** | *0* | **5** | *1* | **4** | *1.25* |
| Very unimportant | Very important | **5** | *1* | **5** | *1* | **4** | *1.25* | **5** | *1* | **4** | *1* | **4** | *1* | **5** | *1* | **5** | *0.5* | **4** | *0.5* | **5** | *1.25* | **4** | *1.5* | **4** | *1.5* | **5** | *0.75* | **4** | *1* | **4.5** | *1* | **5** | *1* |
| The equipment I need to perform the Sepsis Six does NOT work or works poorly | The equipment I need to perform the Sepsis Six DOES work well | **5** | *1* | **4** | *1* | **5** | *2* | **5** | *1* | **5** | *2* | **5** | *1* | **4.5** | *1* | **5** | *1* | **4** | *0.5* | **4** | *1.25* | **4** | *2* | **5** | *1* | **5** | *1* | **5** | *0.5* | **5** | *1* | **4.5** | *1* |
| Very unimportant | Very important | **5** | *1* | **5** | *1* | **5** | *1* | **5** | *1* | **4** | *2* | **4** | *1* | **5** | *1* | **5** | *1* | **4** | *1.5* | **5** | *1* | **4** | *2* | **4** | *1* | **5** | *0* | **4** | *1.5* | **4.5** | *1* | **5** | *0.25* |
| I do NOT intend to continue to perform the Sepsis Six on septic patients | I DO intend to continue to perform the Sepsis Six on septic patients | **5** | *0* | **5** | *0* | **5** | *0* | **5** | *0* | **5** | *0* | **5** | *0* | **5** | *0* | **5** | *0* | **5** | *0* | **5** | *0* | **5** | *0* | **5** | *0* | **5** | *0* | **5** | *0* | **5** | *0* | **5** | *0* |
| Very unimportant | Very important | **5** | *1* | **5** | *0.75* | **5** | *1* | **5** | *0.5* | **5** | *1* | **5** | *1* | **5** | *0* | **5** | *0.5* | **4** | *1* | **5** | *0* | **4** | *2* | **5** | *1* | **5** | *0* | **5** | *1* | **5** | *1* | **5** | *0* |
| Septic patients are RARELY managed in an appropriate location | Septic patients are ALWAYS managed in an appropriate location | **3** | *1* | **3** | *1* | **3** | *2* | **3** | *1* | **3** | *1* | **3** | *1* | **3** | *1* | **3** | *1* | **3** | *0.5* | **3** | *1* | **3** | *1* | **3** | *1* | **3** | *1* | **2** | *1* | **4** | *1* | **3** | *1* |
| Very unimportant | Very important | **5** | *1* | **5** | *1* | **5** | *1* | **5** | *1* | **5** | *1* | **4** | *1* | **5** | *1* | **5** | *0.5* | **4** | *1.5* | **5** | *1* | **4** | *1* | **5** | *1* | **5** | *0.75* | **5** | *1* | **4** | *1* | **5** | *1* |
| Performing the steps in the Sepsis Six does NOT improve patient outcomes | Performing the steps in the Sepsis Six DOES improve patient outcomes | **5** | *0* | **5** | *0* | **5** | *0* | **5** | *0* | **5** | *0* | **5** | *0.75* | **5** | *0* | **5** | *0* | **5** | *0.5* | **5** | *0* | **5** | *0.5* | **5** | *1* | **5** | *0* | **5** | *0.5* | **5** | *0* | **5** | *0* |
| Very unimportant | Very important | **5** | *1* | **5** | *0.75* | **5** | *1* | **5** | *1* | **4** | *1* | **5** | *1* | **5** | *0* | **5** | *1* | **5** | *1* | **5** | *0* | **4** | *1.5* | **5** | *1* | **5** | *0* | **4** | *1* | **5** | *1* | **5** | *0* |
| Sepsis Six performance at this hospital will NOT improve | Sepsis Six performance this this hospital WILL improve | **4** | *1* | **4** | *1.75* | **5** | *1* | **4** | *1* | **4** | *2* | **4** | *0.75* | **5** | *1* | **4** | *0.5* | **4** | *1.5* | **5** | *1.25* | **5** | *1.5* | **4** | *1* | **5** | *0.75* | **4** | *2* | **4** | *0.25* | **5** | *1* |
| Very unimportant | Very important | **5** | *1* | **5** | *1* | **5** | *1* | **5** | *1* | **4** | *2* | **4** | *1* | **5** | *0* | **4** | *1.5* | **4** | *1* | **5** | *1* | **4** | *1.5* | **5** | *1* | **5** | *0* | **4** | *1.5* | **4** | *1.25* | **5** | *0* |
| Overall, the RISKS of performing the Sepsis Six outweigh the benefits | Overall, the BENEFITS of performing the Sepsis Six outweigh the risks | **5** | *1* | **5** | *1* | **5** | *1* | **5** | *1* | **5** | *1* | **5** | *1* | **5** | *0* | **5** | *0.5* | **5** | *1* | **5** | *1* | **5** | *1* | **4** | *1* | **5** | *0* | **5** | *0.5* | **5** | *1* | **5** | *1* |
| Very unimportant | Very important | **5** | *1* | **5** | *1* | **5** | *1* | **5** | *1* | **4** | *1* | **4.5** | *1* | **5** | *0.75* | **5** | *1* | **4** | *1* | **5** | *1* | **4** | *1.5* | **5** | *1* | **5** | *0* | **4** | *1.5* | **4.5** | *1* | **5** | *0* |
| I am UNLIKELY to complete all steps of the Sepsis Six if I think the patient is well | I am LIKELY to complete all steps of the Sepsis Six even if I think the patient is well | **4** | *1* | **4** | *1* | **4** | *1.25* | **3** | *2* | **3** | *2* | **3.5** | *2* | **4** | *2* | **4** | *1.5* | **3** | *2* | **4** | *2* | **3** | *1* | **4** | *1* | **4** | *1.75* | **2** | *2* | **3.5** | *2.25* | **4** | *1* |
| Very unimportant | Very important | **4** | *2* | **4** | *1* | **4** | *2* | **4** | *2* | **4** | *2* | **4** | *2* | **4.5** | *1* | **4** | *0.5* | **4** | *1* | **5** | *1* | **3** | *2* | **4** | *2* | **4.5** | *1* | **3** | *1* | **4** | *2* | **4** | *2* |
| The RISKS of performing the Sepsis Six outweigh the benefits in CERTAIN patient groups | The BENEFITS of performing the Sepsis Six outweigh the risks in ALL patient groups | **4** | *2* | **4** | *2* | **4** | *3* | **4** | *2* | **4** | *3* | **4** | *2.75* | **4** | *2* | **4** | *2* | **2** | *1.5* | **4** | *2* | **3** | *2.5* | **3** | *2* | **5** | *0* | **5** | *2* | **4** | *1.25* | **4** | *1* |
| Very unimportant | Very important | **4** | *1* | **5** | *1* | **4** | *1.25* | **4** | *1* | **4** | *1* | **4** | *1.75* | **5** | *1* | **4** | *1* | **4** | *1.5* | **5** | *1* | **4** | *1.5* | **4** | *1.5* | **5** | *0.75* | **4** | *0* | **4** | *1* | **4.5** | *1.25* |
| There is POOR teamwork when looking after septic patients | There is GOOD teamwork when looking after septic patients | **4** | *2* | **4** | *1* | **4** | *1.25* | **4** | *1.5* | **3** | *1* | **4** | *2* | **4** | *1* | **4** | *0.5* | **4** | *1.5* | **4** | *1* | **3** | *1.5* | **4** | *1* | **4** | *1* | **4** | *1* | **4** | *2* | **4.5** | *1* |
| Very unimportant | Very important | **5** | *1* | **5** | *1* | **5** | *1* | **5** | *1* | **4** | *1* | **4** | *1* | **5** | *0* | **5** | *0.5* | **4** | *1* | **5** | *0* | **4** | *1* | **4** | *1* | **5** | *0.75* | **4** | *1* | **4.5** | *1* | **5** | *0.25* |
| Early and regular reassessment of patients requiring the Sepsis Six has NO effect on outcomes | Early and regular reassessment of patients requiring the Sepsis Six gives the BEST outcomes | **5** | *0* | **5** | *0* | **5** | *0* | **5** | *0* | **5** | *0* | **5** | *0* | **5** | *0* | **5** | *0* | **5** | *1* | **5** | *0* | **5** | *0* | **5** | *0* | **5** | *0* | **4** | *1* | **5** | *0* | **5** | *0* |
| Very unimportant | Very important | **5** | *1* | **5** | *1* | **5** | *1* | **5** | *1* | **4** | *1* | **5** | *1* | **5** | *0* | **5** | *1* | **4** | *1* | **5** | *0* | **5** | *1.5* | **5** | *1* | **5** | *0* | **4** | *0.5* | **5** | *1* | **5** | *0* |
| The hospital is NOT formally rewarded for good Sepsis Six performance | The hospital IS formally rewarded for good Sepsis Six performance | **3** | *1* | **3** | *1* | **3** | *2* | **3** | *1* | **3** | *1* | **3** | *1* | **3** | *1* | **4** | *1.5* | **3** | *1* | **3** | *1* | **3** | *1* | **3** | *1* | **3** | *2* | **4** | *1* | **3** | *1.25* | **3** | *0.5* |
| Very unimportant | Very important | **4** | *2* | **4** | *2* | **3** | *2* | **3** | *1* | **3** | *1* | **3** | *1* | **4** | *2* | **4** | *0.5* | **3** | *1.5* | **4.5** | *1.25* | **3** | *1.5* | **3** | *1* | **3.5** | *2* | **3** | *1* | **3** | *0* | **3.5** | *2* |
| I am NOT confident performing the Sepsis Six | I AM confident performing the Sepsis Six | **5** | *0* | **5** | *0* | **5** | *0* | **5** | *0.5* | **5** | *0* | **5** | *0* | **5** | *1* | **5** | *0* | **5** | *0* | **5** | *0* | **5** | *0* | **5** | *0* | **5** | *0* | **5** | *0* | **5** | *0* | **4** | *1* |
| Very unimportant | Very important | **5** | *1* | **5** | *0* | **5** | *1* | **5** | *1* | **4** | *1* | **5** | *1* | **5** | *0* | **5** | *0.5* | **4** | *1.5* | **5** | *0* | **4** | *2* | **5** | *1* | **5** | *0* | **4** | *0.5* | **5** | *0.25* | **5** | *0* |
| I do NOT prioritise performing the Sepsis Six on a septic patient over other tasks | I DO prioritise performing the Sepsis Six on a septic patient over other tasks | **5** | *1* | **4.5** | *1* | **5** | *1* | **5** | *0* | **4** | *1* | **5** | *1* | **5** | *1* | **5** | *1* | **5** | *1* | **4** | *1* | **4** | *1* | **5** | *1* | **5** | *0.75* | **5** | *1* | **5** | *0* | **5** | *0* |
| Very unimportant | Very important | **5** | *1* | **5** | *1* | **4** | *1* | **5** | *1* | **4** | *1* | **4.5** | *1* | **5** | *1* | **5** | *0* | **4** | *2* | **5** | *1* | **4** | *1* | **4** | *1* | **5** | *0* | **5** | *1* | **5** | *1* | **5** | *0* |
| Some of the steps in the Sepsis Six are MORE DIFFICULT to perform than others | The steps in the Sepsis Six are EQUALLY EASY OR DIFFICULT to perform | **4** | *3* | **4** | *2.75* | **3** | *3* | **4** | *2* | **3** | *3* | **3.5** | *3* | **4** | *2* | **2** | *3* | **2** | *3.5* | **4.5** | *2* | **3** | *2* | **3** | *3.5* | **4** | *3.5* | **5** | *2* | **4** | *2.25* | **3** | *1.25* |
| Very unimportant | Very important | **4** | *1* | **5** | *1* | **4** | *1.25* | **4** | *1.5* | **4** | *1* | **4** | *1.5* | **5** | *1* | **4** | *0.5* | **4** | *0.5* | **5** | *0.25* | **4** | *1* | **4** | *1* | **4.5** | *1* | **4** | *1.5* | **4** | *1.25* | **5** | *1* |
| SOME steps in the Sepsis Six are more or less important than others | ALL steps in the Sepsis Six are equally important | **4** | *2* | **4** | *3* | **4.5** | *2* | **4** | *3* | **3** | *3* | **3.5** | *3* | **5** | *1* | **2** | *2* | **2** | *2.5* | **5** | *1* | **3** | *1.5* | **4** | *2* | **5** | *0* | **5** | *2.5* | **2.5** | *3* | **4** | *1* |
| Very unimportant | Very important | **4** | *1* | **4.5** | *1* | **4** | *2* | **4** | *1* | **4** | *2* | **4** | *2* | **5** | *1* | **4** | *0.5* | **4** | *1.5* | **5** | *1* | **4** | *1.5* | **4** | *1.5* | **4.5** | *1* | **4** | *2* | **4** | *1* | **5** | *1* |
| There is POOR communication between members of the team looking after septic patients | There is GOOD communication between members of the team looking after septic patients | **4** | *2* | **4** | *2* | **4** | *2* | **4** | *1.5* | **3** | *2* | **4** | *1* | **4** | *1* | **3** | *1* | **4** | *1* | **4** | *1.25* | **3** | *2* | **4** | *1* | **4.5** | *1.75* | **4** | *1.5* | **3.5** | *1* | **4** | *1* |
| Very unimportant | Very important | **5** | *1* | **5** | *1* | **5** | *1* | **5** | *1* | **4** | *1* | **4** | *1* | **5** | *1* | **5** | *0.5* | **4** | *0.5* | **5** | *1* | **4** | *1* | **5** | *1* | **5** | *1* | **4** | *0.5* | **4.5** | *1* | **5** | *0.25* |
| We provide POOR sepsis care at this hospital | We provide GOOD sepsis care at this hospital | **4** | *1* | **4** | *2* | **4** | *1.25* | **4** | *1* | **3** | *1* | **4** | *1* | **4** | *1.75* | **4** | *1* | **4** | *1* | **4** | *1.25* | **3** | *1* | **4** | *0.5* | **4** | *1.75* | **3** | *1.5* | **4** | *1* | **4** | *0.25* |
| Very unimportant | Very important | **4** | *1* | **5** | *1* | **4** | *1* | **4** | *1* | **4** | *2* | **4** | *1* | **5** | *1* | **4** | *1.5* | **4** | *1.5* | **5** | *1* | **4** | *2* | **4** | *1* | **5** | *1* | **4** | *1* | **4** | *0.25* | **5** | *1* |
| I do NOT have a time-based goal for completing the Sepsis Six on septic patients | My goal is to complete the Sepsis Six within an HOUR on all septic patients | **5** | *0* | **5** | *0* | **5** | *0.25* | **5** | *0* | **5** | *1* | **5** | *0* | **5** | *0* | **5** | *0* | **5** | *0.5* | **5** | *0* | **5** | *1* | **5** | *0* | **5** | *0* | **5** | *0.5* | **5** | *0* | **5** | *0* |
| Very unimportant | Very important | **5** | *1* | **5** | *0* | **5** | *1* | **5** | *0.5* | **4** | *1* | **5** | *1* | **5** | *0* | **5** | *0* | **4** | *1* | **5** | *0* | **4** | *0.5* | **5** | *0.5* | **5** | *0* | **5** | *1* | **5** | *0.25* | **5** | *0* |
| Increasing Sepsis Six performance will NOT improve patient care | Increasing Sepsis Six performance WILL improve patient care | **5** | *0* | **5** | *0* | **5** | *0* | **5** | *0* | **5** | *1* | **5** | *0* | **5** | *0* | **5** | *0* | **5** | *0* | **5** | *0* | **5** | *1* | **5** | *0* | **5** | *0* | **5** | *0.5* | **5** | *0* | **5** | *0* |
| Very unimportant | Very important | **5** | *1* | **5** | *1* | **5** | *1* | **5** | *1* | **4** | *1* | **5** | *1* | **5** | *0* | **5** | *0.5* | **4** | *1* | **5** | *0* | **4** | *1* | **5** | *1* | **5** | *0* | **4** | *0.5* | **5** | *1* | **5** | *0* |
| Individuals are NOT formally rewarded for good Sepsis Six performance | Individuals ARE formally rewarded for good Sepsis Six performance | **2** | *2* | **2** | *2* | **2** | *2* | **1** | *2* | **2** | *2* | **1** | *1.75* | **2** | *2* | **3** | *2* | **1** | *1* | **2** | *2* | **2** | *2* | **2** | *2* | **2** | *3.5* | **1** | *0* | **1** | *0.25* | **2.5** | *3* |
| Very unimportant | Very important | **3** | *2* | **4** | *2* | **3** | *2* | **3** | *2* | **4** | *1* | **3** | *2* | **4** | *2* | **4** | *1* | **3** | *1* | **4** | *2* | **4** | *1* | **3** | *1.5* | **4** | *2.75* | **3** | *1* | **3** | *2* | **4** | *2.5* |
| I do NOT feel anxious/stressed when treating septic patients | I DO feel anxious/stressed when treating septic patients | **2** | *3* | **2** | *2* | **2.5** | *3* | **3** | *2* | **2** | *2* | **3** | *2.75* | **2** | *3* | **2** | *1.5* | **3** | *1.5* | **2** | *2* | **1** | *2* | **2** | *2.5* | **3** | *2.75* | **2** | *1* | **3.5** | *1.5* | **2.5** | *1.25* |
| Very unimportant | Very important | **4** | *2* | **4** | *1* | **3** | *2* | **4** | *1.5* | **3** | *1* | **3** | *1* | **5** | *2* | **4** | *0* | **3** | *1.5* | **5** | *1* | **3** | *1.5* | **3** | *1.5* | **4** | *2* | **3** | *2* | **3.5** | *1.25* | **4** | *2* |
